# A ribosomal marker-based metataxonomic framework for environmental surveillance of nematodes of public health importance

**DOI:** 10.1371/journal.pone.0348689

**Published:** 2026-08-28

**Authors:** Juan P. Zuluaga, Katherine Bedoya-Urrego, Juan F. Alzate

**Affiliations:** 1 Escuela de Microbiología, Universidad de Antioquia, Medellín, Colombia; 2 Centro de Secuenciación Genómica (CNSG), Sede de Investigación Universitaria (SIU), Universidad de Antioquia, Medellín, Colombia; 3 Departamento de Microbiología y Parasitología, Facultad de Medicina, Universidad de Antioquia, Medellín, Colombia; Quinnipiac University, UNITED STATES OF AMERICA

## Abstract

Metataxonomic analysis targeting the V4 region of the 18S rDNA gene, combined with molecular phylogenetic inference, was applied to detect nematode DNA of public health relevance in environmental matrices. A total of 25 mOTUs corresponding to six nematode taxa were detected in environmental samples from the Andean region of Colombia. Analysis of 12 water and sludge samples from wastewater treatment plants, 5 artisanal agricultural bioinputs, and 3 food samples revealed multiple species of public health significance: *Trichuris trichiura*, *Enterobius vermicularis*, *Ascaris* spp., and *Necator americanus.* We also confirmed zoonotic species, including *Angiostrongylus cantonensis* and *Trichinella* spp*.* These findings demonstrate that combining metataxonomics with molecular phylogeny provides a scalable molecular framework for the environmental surveillance of parasitic nematodes, overcoming the limitations of traditional morphological identification methods. This approach offers a replicable model for strengthening control and monitoring programs for parasitism in human populations.

## Introduction

Nematodes are ubiquitous metazoans found in terrestrial and aquatic habitats, parasitizing plants and animals including humans [1,2]. Approximately 30,000 species have been described, though total diversity may approach one million [3–5]. They exhibit remarkable trophic diversity, feeding on bacteria, fungi, algae, protozoa, and other nematodes, or living as facultative or obligate parasites [5,6]. In Colombia, nematode infections pose a significant public health problem, particularly among children [7]. Anthelmintic resistance is well established in many animal-infective helminths, where intensive drug use has selected for resistant populations and compromised veterinary control programs [8,9]. In human helminths, by contrast, resistance is not yet firmly established: reduced or suboptimal treatment responses have been reported, but the supporting evidence remains limited [10]. This distinction nonetheless reinforces the need for more sensitive molecular tools to monitor parasite circulation and treatment efficacy.

Nematode species identification typically relies on morphological characteristics of adults, larvae, and eggs [11,12]. However, classical methods often produce nonspecific identifications, underestimating parasite circulation [6,12,13]. For instance, adult and larvae morphological similarities in mouthpart, esophagus and tail structures can obscure distinctions between species, and variations in body length may not adequately reflect species differences due to overlapping size ranges. Taxonomic resolution is frequently limited to genus level; for example, microscopic examination of hookworm eggs cannot differentiate *Necator americanus* from *Ancylostoma duodenale*, nor can it distinguish these human hookworms from the morphologically indistinguishable eggs of animal and zoonotic hookworm species such as *Ancylostoma ceylanicum* and *Ancylostoma caninum* [14]. Despite their ecological and physiological diversity, nematodes conserved morphology and small size provide few phylogenetically informative characters, many showing convergent evolution [6]. In environmental samples, clinically relevant nematode eggs may be misidentified as Acari (mite) eggs due to morphological similarities, potentially leading to diagnostic errors [15].

Conventional diagnostic methods cannot adequately detect parasitic nematode diversity [6]. In previous studies, metataxonomic approaches targeting ribosomal rDNA regions have demonstrated high sensitivity and specificity for cryptic or closely related species [16–18]. Though taxonomic resolution may be limited to genus level, metataxonomics offers reproducibility, scalability, automation, and cost-effectiveness [17], and facilitates parasite detection in complex environmental matrices [18]. While widely applied in prokaryotes [19–21], nematode metataxonomic studies remain emerging [3,22]. In parallel, wastewater- and soil-based environmental surveillance is increasingly being explored as a complementary strategy for monitoring soil-transmitted helminths in endemic settings [23].

Accurate detection of parasitic nematodes in environmental matrices is critical for environmental surveillance and public health monitoring yet remains constrained by limitations in sensitivity and taxonomic resolution. To address these challenges, this study implements metataxonomics coupled with phylogenetic analyses to detect nematodes of public health importance in environmental samples from the Andean region of Colombia.

We hypothesize that integrating metataxonomic profiling of the 18S rDNA V4 region with phylogenetic inference based on concatenated 18S–28S reference sequences enhances detection sensitivity and provides a versatile framework for identifying nematodes across heterogeneous environmental matrices. This approach enables robust taxonomic assignment and detection of morphologically cryptic and environmentally persistent taxa [17,22], while supporting the development of scalable molecular protocols for parasite surveillance within wastewater-based epidemiology and One Health frameworks.

## Materials and methods

### Sample collection and DNA extraction

Environmental samples were collected from four wastewater treatment plants (WWTPs), as well as from artisanal bioinputs and food sources. A total of 12 samples were obtained from the WWTPs. The four plants collectively serve an estimated population of 5.5 million inhabitants and are located at elevations ranging from 1,080 m above sea level (m a.s.l.) in Cali to 2,175 m a.s.l. San Fernando and Aguas Claras are located in the metropolitan area of Medellín (Aburrá Valley), at 1,550 and 1,300 m a.s.l., respectively, and serve the city of Medellín and neighbouring municipalities [18]. San Fernando treats an influent of ~1.3 m^3^/s of municipal wastewater by activated sludge, serving 700,000 inhabitants and generating 85 tons of biosolids per day. Aguas Claras treats ~5 m^3^/s by activated sludge, serving 2,200,000 inhabitants and generating 126 tons of biosolids per day. Cañaveralejo, in the city of Cali, applies advanced primary treatment to ~4 m^3^/s of municipal wastewater, serves 2,600,000 inhabitants, and generates 60 tons of biosolids per day. El Retiro is a smaller rural plant in the municipality of El Retiro (Antioquia) that treats ~0.6 m^3^/s by activated sludge, serves 20,000 inhabitants, and generates 2 tons of biosolids per day [18]. Sampling design, sample types, and collection years for all sites are summarized in S1 Table.

Five commercial bioinput samples, solid and liquid organic fertilizers marketed for agricultural use (three from Medellín and two from Cali) and three food samples from the Aburrá Valley, comprising fresh produce intended for raw consumption and a processed cereal-based product, were analysed. As these were commercially available products, their specific formulations and brands are proprietary and are not disclosed. All samples were processed using the same extraction protocol described above, during 2024 and 2023, respectively.

Prior to extraction, samples were homogenized using sterile instruments. DNA extraction was performed using the QIAGEN DNeasy PowerSoil Kit, processing 200 mg aliquots of environmental samples. The concentration and quality of purified DNA was determined by UV spectrophotometry, and samples were cryopreserved at −20 °C until use for PCR amplification [18].

### Metataxonomic analysis

For DNA amplification, degenerate primers were used, designed to target the hypervariable V4 region of the eukaryotic 18S ribosomal gene (18S rDNA). The forward primer corresponded to the sequence 18S-V4Fw: (CCAGCAGCCGCGGTAATTCC) [24], the reverse primer used was 18S-V4Rev (RCYTTCGYYCTTGATTRA). These primers were successfully applied to the same sample set [18], in a study focused on protists. PCR amplification, genomic library preparation, and high-throughput sequencing services were outsourced to Macrogen Inc. (Seoul, South Korea), using the Illumina MiSeq platform configured for 300 bp paired-end reads.

Bioinformatic processing of the obtained sequences was performed using MOTHUR (v.1.44.3), following the protocol described by Rozo-Montoya et al. (2023). This process included the merging of paired-end reads, filtering of sequences containing ambiguous bases or shorter than 300 bp, removal of sequences with homopolymers longer than eight bases, clustering by sequence similarity, detection and removal of chimeric sequences, and construction of molecular operational taxonomic units (mOTUs) using a 97% similarity threshold*.*

Preliminary taxonomic assignment of the mOTUs was performed using the *classify.seqs* algorithm implemented in MOTHUR, with the SILVA (v.138) database serving as the reference [25]. Only mOTUs classified as eukaryotic were selected for subsequent BLASTn analysis [26], and phylogenetic studies. Sequencing quality indicators, including the number of high-quality sequences, mOTUs counts, and coverage estimators, were calculated using the *summary.single* command in MOTHUR. The raw 18S rDNA amplicon sequencing data used in this study are publicly available in the NCBI Sequence Read Archive (SRA) under BioProject accessions numbers PRJNA976754 and PRJNA1516045 (https://www.ncbi.nlm.nih.gov/sra/?term=PRJNA976754).

### Species selection and bioinformatic processing

For this analysis, nematodes of public health importance were selected, including human-parasitic and zoonotic species [27,28]. In total, the reference database incorporated 49 species represented across 27 genera.

To obtain complete and high-quality rDNA sequences, nematode reference genomes of target species were downloaded from the NCBI database using its available datasets and a bioinformatic routine optimized for batch downloading. When no reference genome was available for a species of interest, complementary RNA-seq data were obtained from the NCBI Sequence Read Archive (SRA), followed by the extraction of contigs containing rDNA gene sequences using the Trinity program [29]. Neither genomic nor RNA-seq data were available for *Strongyloides ransomi*, instead partial 18S rDNA sequences (AB453327, OP288111) were included.

From the reference genomes, an annotation and extraction strategy was implemented to specifically retrieve the 18S and 28S ribosomal regions using Barrnap (v0.9) [30], combined with an auxiliary bioinformatic pipeline. The annotated and extracted sequences underwent quality screening to remove ambiguous, fragmented, or incomplete sequences. Only sequences with a combined length ≥ 1,400 bp across the 18S and 28S regions were retained. From the filtered set, a single consensus sequence with the highest overall quality and length was selected.

The resulting rDNA sequences were subjected to inspection and curation, including verification of orientation and length, format standardization, and consistent identifier assignment to ensure proper handling in subsequent analyses with SeqKit (v2.10.1) [31]. The 18S and 28S sequences were aligned separately using MAFFT (v7.215) [32], and the resulting alignments were inspected in AliView [33]. Finally, both alignment sets were concatenated using FASconCAT-G (v1.06.1) [34].

### Phylogenetic analysis using 18S and 28S rDNA markers

To identify the putative nematode molecular operational taxonomic units (mOTUs) of interest in this study, a nucleotide identity–based search strategy was employed using BLASTn. For this purpose, the mOTUs generated with MOTHUR were compared against a local reference database containing concatenated 18S and 28S rDNA regions. mOTUs showing ≥95% sequence identity, representing the degree of match between aligned sequences, and a BLAST score ≥500, which reflects the overall quality and significance of the alignment according to the BLASTn algorithm, were considered valid candidates for phylogenetic analysis.

Reference sequences of 18S and 28S rDNA, together with the V4 region of the 18S rDNA from the putative mOTUs identified through BLASTn, were aligned using MAFFT. The resulting alignment was manually inspected to identify and correct potential conflicting regions using AliView. Subsequently, maximum likelihood (ML) phylogenetic trees were constructed with the aligned sequences using IQ-TREE3 [35]. The robustness of branch support was evaluated using a dual approach: Ultrafast Bootstrap (UFBoot) and the Approximate Likelihood Ratio Test (aLRT), both computed with 5,000 replicates.

### Data management and presentation

Basic descriptive statistical analyses were performed using custom routines implemented in Python, including calculations of taxa presence–absence, occurrence frequencies, and genus- and species-level richness across samples and sampling sites. These summaries were used to support comparative interpretation of nematode diversity and distribution patterns.

Phylogenetic trees were visualized and edited using FigTree (v1.4.4) [36], where tree topologies were examined and clades of interest were selectively collapsed to enhance interpretability. Branch coloring and additional graphical refinements were subsequently applied using image editing software.

For graphical representation of spatial patterns, regional distribution maps were generated using QGIS (v3.40.11) [37]. Terrain relief was derived from the digital elevation model distributed with the ALOS PALSAR High-Resolution Radiometrically Terrain-Corrected product [38] which is a geoid-corrected, resampled copy of the SRTM DEM. Administrative boundaries (departments and municipalities) were obtained from the open-data repository of the Instituto Geográfico Agustín Codazzi [39]. Heatmaps summarizing taxa occurrence and relative abundance patterns were produced in R (v4.3.1) using the dplyr and ggplot2 packages.

All author-generated code used in this study, including the metataxonomic pipeline and the Python and R analysis scripts, is provided in S1 File.

## Results

### Construction of mOTUs and sequence processing

In each amplicon library, a minimum of 109,906 pairs of raw reads were obtained, with a maximum of 334,064 reads per sample. After merging and quality-filtering processes, the number of retained high-quality sequences ranged from 37,705–77,967. The number of molecular operational taxonomic units (mOTUs) detected across samples varied between 374 and 866. The coverage index ranged from 99.4% to 99.7%, indicating a high level of sampling of the expected theoretical diversity.

### Construction of a local database from Genomes

A total of 63 genomes representing 49 nematode species across 27 genera were analysed from the NCBI database. Genome sizes ranged from 42.5 to 656.4 Mb (median: 111.8 Mb).

Assembly continuity (N50) varied from 1.2 kb to 110.8 Mb (median: 1,095.2 kb), while fragmentation ranged from 2 to 167,310 contigs (median: 651). GC content ranged between 21.30% and 47.96% (mean: 36.68%). Most assemblies showed low levels of ambiguous bases (median: 0.33%), although higher values were observed in *Oesophagostomum dentatum*, *Romanomermis culicivorax*, and *Ancylostoma ceylanicum.*

The extraction of rDNA sequences using Barrnap was highly effective, achieving a success rate of 98.4% (62 out of 63 analyzed genomes). The only exception corresponded to *Enterobius vermicularis* (GCA_900576705), for which the software failed to identify ribosomal regions; thus, the dataset was complemented with nucleotide sequences obtained through partial RNA-seq annotation from assembly SRA_ERR310935 and Sanger sequences (FR687850, AF182295, JF934731, LC416069). A total of 14,875 rDNA sequences were recovered from genomes using this methodology: 6,276 corresponding to 18S and 8,599 to 28S. The concatenated consensus sequences (18S + 28S) ranged in length from 1,448–8,682 bp, with an average length of 4,925 bp, while the percentage of ambiguous nucleotides remained ≤ 0.03% in all cases.

### Taxonomic assignment of mOTUs by phylogenetic inference of rDNA

**Fig 1:**
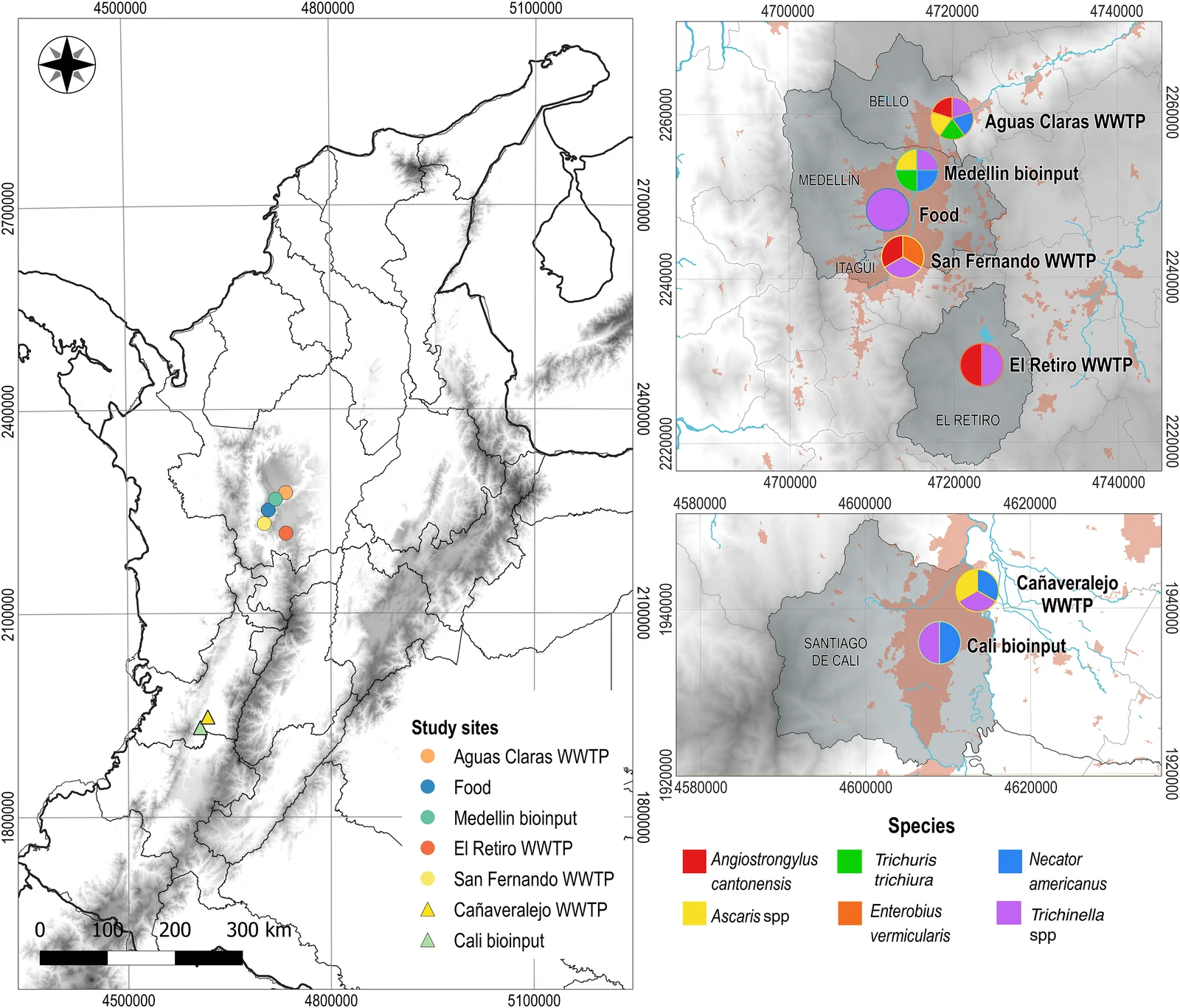
National and regional map of the Andean zone of Colombia showing all sampling locations included in the study. Coloured markers indicate the sites where nematode DNA was detected in wastewater treatment plants (WWTPs), bioinputs, and food items collected between 2021 and 2024. The figure illustrates the geographic distribution of nematode detections across urban and peri-urban areas and identifies areas of potential interest for epidemiological monitoring. Map generated by the authors in QGIS v3.40.11; terrain relief from ASF DAAC; administrative boundaries from IGAC.

The initial analysis performed with MOTHUR generated a total of 16,045 mOTUs. Subsequently, the nucleotide identity search using BLASTn against the local reference database enabled the recovery of 933 hits that met the established filtering criteria (identity ≥ 95% and score ≥ 500). After removing redundant hits, 292 representative sequences were retained. These candidate sequences were aligned with MAFFT alongside the consensus reference sequences and subjected to phylogenetic inference. The general description of nematode genus per sample is described in Fig 1.

The maximum likelihood phylogenetic analysis was based on the 25 mOTUs retained after filtering. The phylogenetic reconstruction of the nematodes of interest in this study showed high levels of statistical support, with UFBoot and aLRT values ≥ 95% for most clades, both calculated from 5,000 replicates. This degree of confidence allowed the consolidation of well-defined and clearly separated clades, consistent with previously reported phylogenetic structures described by other authors [1,3,5,27]. Although it was necessary to supplement certain lineages not derived from reference genomes, the individual clade supports were sufficiently robust to allow a reliable assignment of the mOTUs included in the analysis.

**Fig 2:**
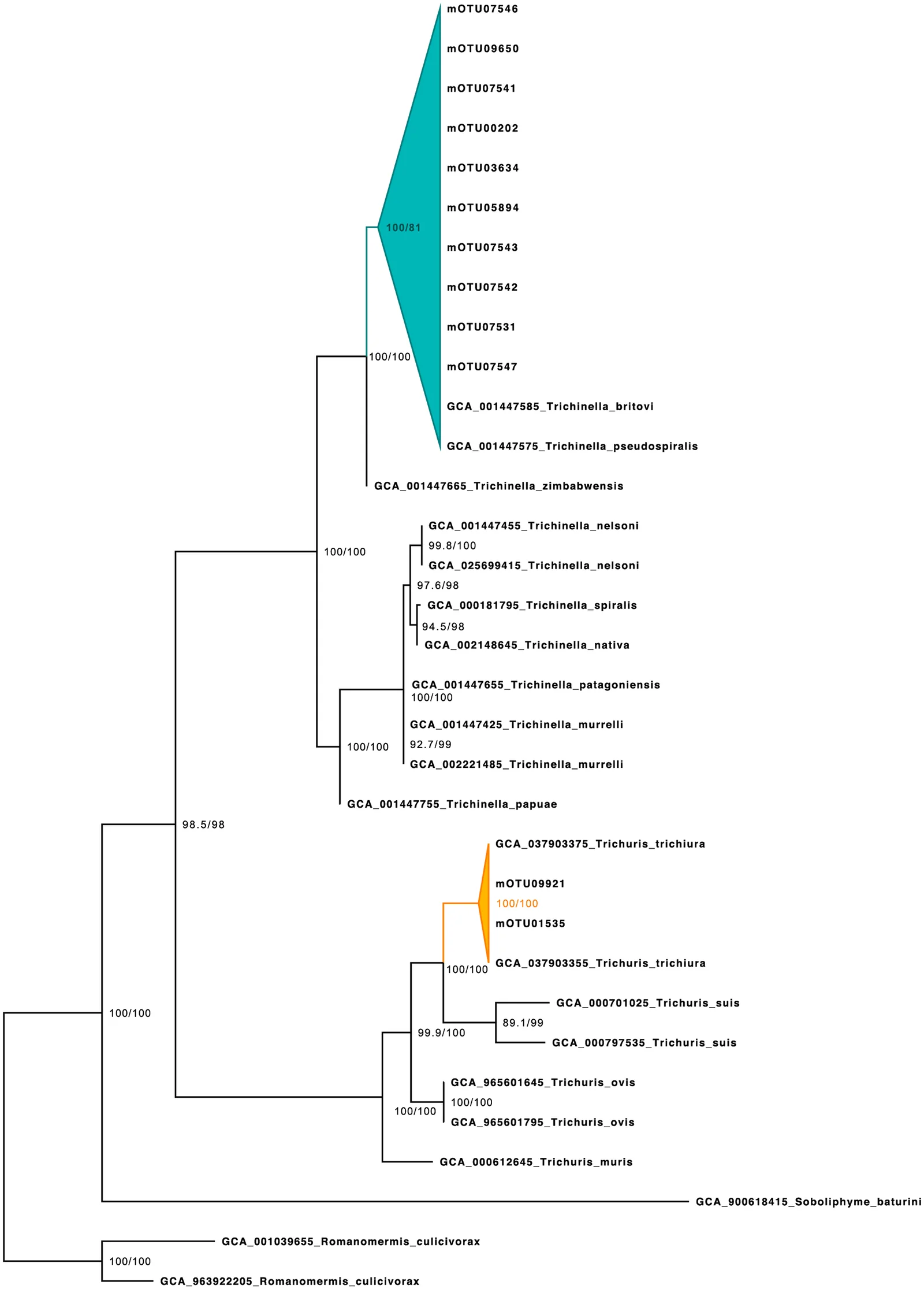
Phylogenetic analysis of Clade I for taxonomic assignment. Maximum-likelihood phylogenetic tree of Clade I, based on concatenated rDNA sequences (18S + 28S). Molecular operational taxonomic units (mOTUs) identified in the samples are labeled on the tree. High support values (UFBoot and aLRT ≥ 95%) demonstrate the reliability of genus-level and, in some cases, species-level assignments. This clade includes nematodes of clinical and zoonotic relevance, supporting the utility of molecular monitoring in environmental matrices.

In Clade I, the presence of *Trichuris trichiura* was confirmed with high phylogenetic support (UFBoot and aLRT ≥ 95%). For *Trichinella* spp. support values were slightly lower but still sufficient to enable reliable genus-level assignment*.* For *Trichinella* spp., phylogenetic analysis revealed clear separation from closely related species, with the mOTUs clustering within the clade comprising *T. britovi* and *T. pseudospiralis* (Fig 2).

**Fig 3:**
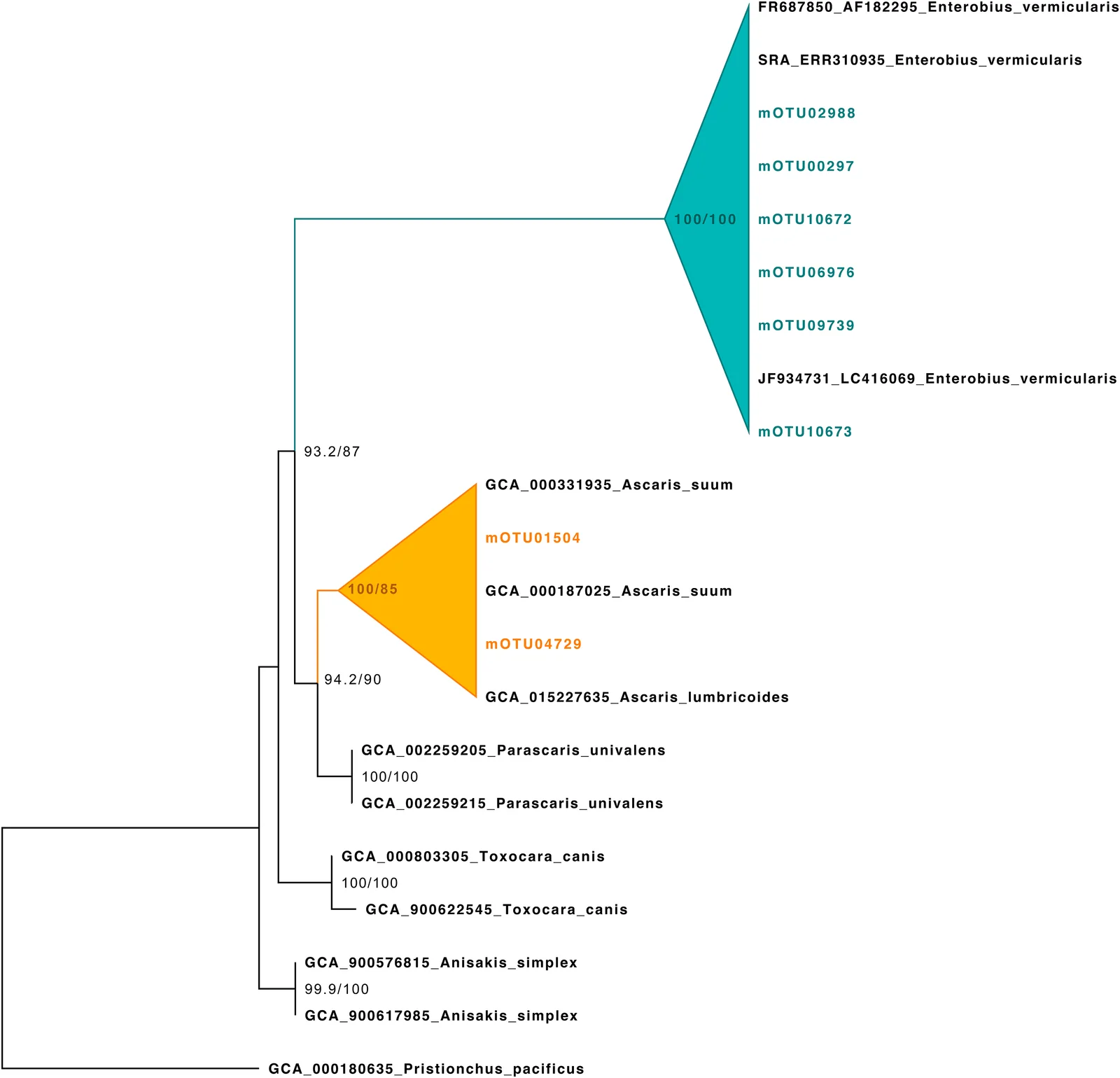
Phylogenetic analysis of Clade III for taxonomic assignment. Maximum-likelihood phylogenetic tree of Clade III, showing the placement of mOTUs identified in the study. The figure highlights intra-clade diversity and reveals phylogenetic relationships among nematodes present in wastewater and bioinput samples. Branch support values indicate the robustness of the assignments and provide confidence in interpreting potential transmission pathways.

In Clade III, *Enterobius vermicularis* was assigned with high confidence (UFBoot and aLRT ≥ 95%) and represented by five mOTUs. For the genus Ascaris, phylogenetic resolution did not allow differentiation between *Ascaris suum* and *A. lumbricoides* due to their close evolutionary relationship (Fig 3).

In Clade IV, no species were confidently detected with support values above the significance threshold (UFBoot and aLRT ≥ 95%). Detailed results for this clade are therefore provided in the supplementary material (S1 Fig).

**Fig 4:**
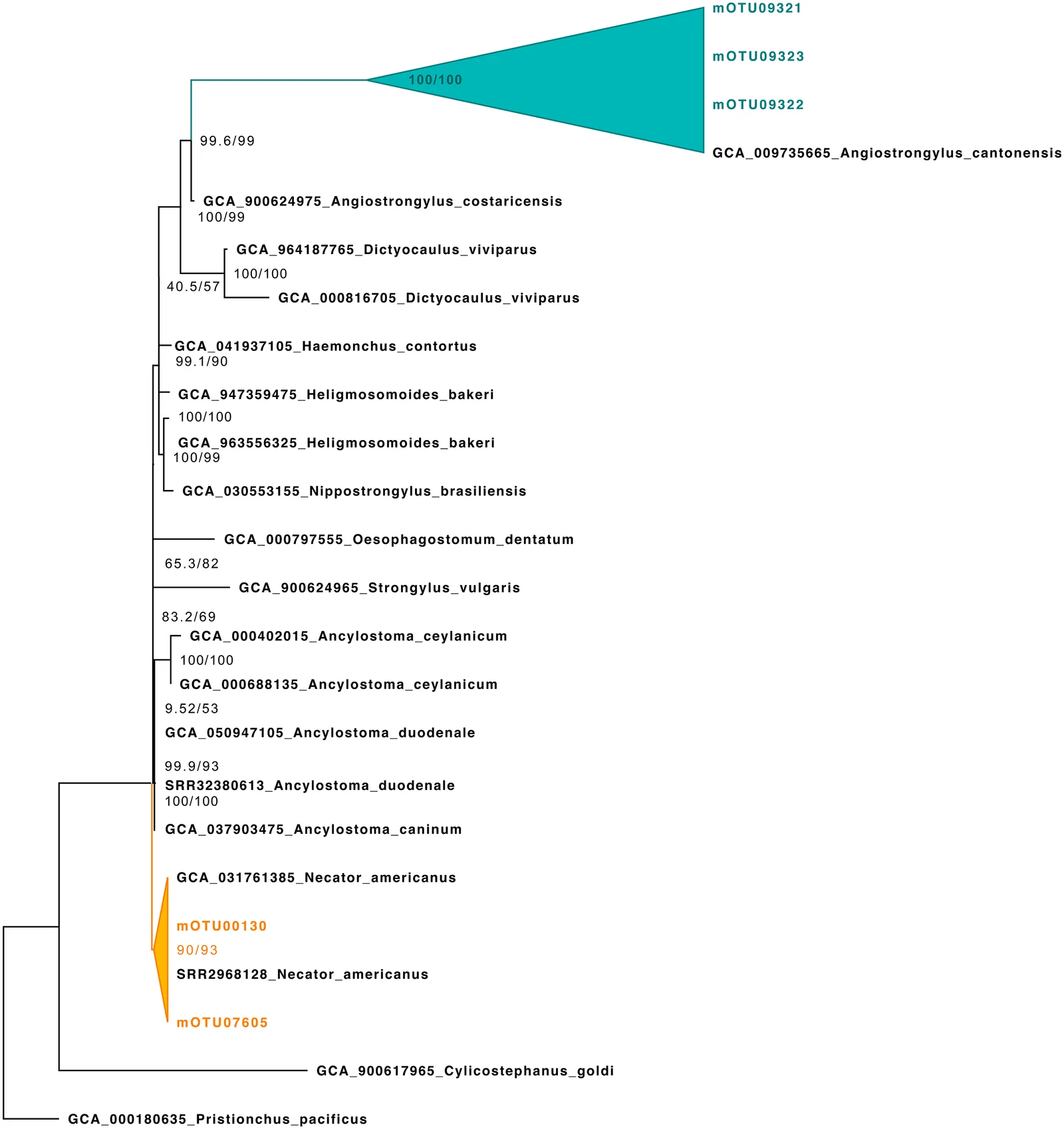
Phylogenetic analysis of Clade V for taxonomic assignment. Maximum-likelihood phylogenetic tree of Clade V, illustrating the placement of mOTUs detected in environmental samples. Support values (UFBoot and aLRT from 5,000 replicates) demonstrate reliable phylogenetic inference. This clade includes nematodes with varying degrees of public health significance, highlighting the importance of environmental surveillance for both human and zoonotic pathogens.

Finally, in Clade V, *Angiostrongylus cantonensis* were assigned with robust support (UFBoot and aLRT ≥ 95%). For *Necator americanus*, support was slightly lower, although clustering within a well-defined clade allowed confirmation of its taxonomic identity (Fig 4).

### Temporal dynamics and distribution of nematode DNA in wastewater treatment plants (WWTPs)

**Fig 5:**
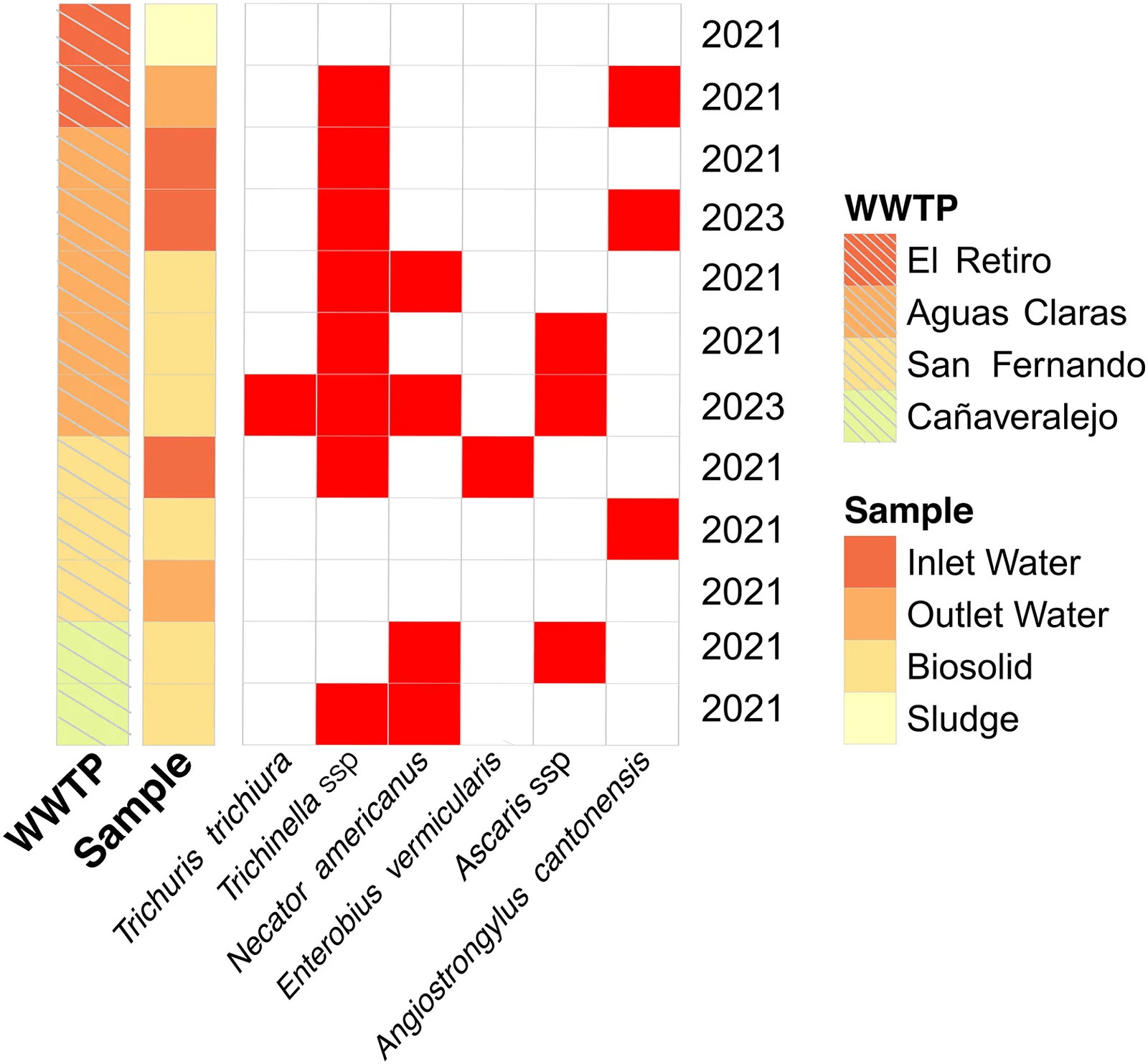
Comparative heatmaps of nematode DNA detection across wastewater treatment plants. Heatmaps illustrate the presence and absence of nematode DNA across all analyzed matrices between 2021 and 2024. Wastewater treatment plants (WWTPs), showing spatial and temporal patterns of detection and identifying facilities that act as persistent reservoirs of nematode genetic material.

Nematode DNA was detected in 10 of the 12 WWTP samples, with defined spatial distribution patterns at each study site (Fig 5). Aguas Claras WWTP, sampled in both 2021 and 2023, showed a consistent longitudinal pattern of detection. In the 2021 influent (sample K7F4002), *Trichinella* spp. was detected. The biosolids from this plant appeared to act as a reservoir: *Necator americanus* and *Trichinella* spp. were recovered from one biosolid sample (K7B2001), whereas *Ascaris* spp. and *Trichinella* spp. were recovered from the other (K7B2002), both collected in 2021. In the 2023 influent (sample M9F4003), *Trichinella* spp. and *Angiostrongylus cantonensis* were identified, while the 2023 biosolids (sample M9B2003) contained *Trichuris trichiura*, *Trichinella* spp., *N. americanus*, and *Ascaris* spp. Overall, *Trichinella* spp. was recovered from every Aguas Claras sample, and geohelminth DNA (*N. americanus* and *Ascaris* spp.) was detected in both sampling years, indicating sustained circulation rather than sporadic contamination (Fig 5, S2 Table).

In San Fernando WWTP, during 2021, the influent water (sample K7F4004) contained *E. vermicularis* and *Trichinella* spp. In the corresponding biosolid (sample K7B3001), *A. cantonensis* was detected, while the effluent showed no detectable nematode DNA (Fig 5, S2 Table).

In Cañaveralejo WWTP (Cali), biosolid analysis revealed the persistence of *Ascaris* spp. and *N. americanus*, in sample K7B1001 and the detection of nematode DNA corresponding to *N. americanus*, and *Trichinella* spp. in sample K7B1002 (Fig 5, S2 Table).

Finally, in El Retiro WWTP, residual sludge (sample K7B4001) showed no detectable nematode DNA, whereas the effluent water (sample K7F4001) contained *Trichinella* spp. and *A. cantonensis*. (Fig 5, S2 Table).

### Presence of nematode DNA in bioinputs and foods

In the analysis of commercial bioinputs, DNA from species of public health relevance was detected in all five samples, each testing positive for *Trichinella* spp. (N8C1001, N8C1002, N8C1003, N8C2001, and N8C2002). Sample N8C1002 exhibited the most diverse parasitic community, additionally testing positive for *Necator americanus*, *Ascaris* spp., and *Trichuris trichiura*. *N. americanus* was also detected in samples N8C1001 and N8C2001.

**Fig 6:**
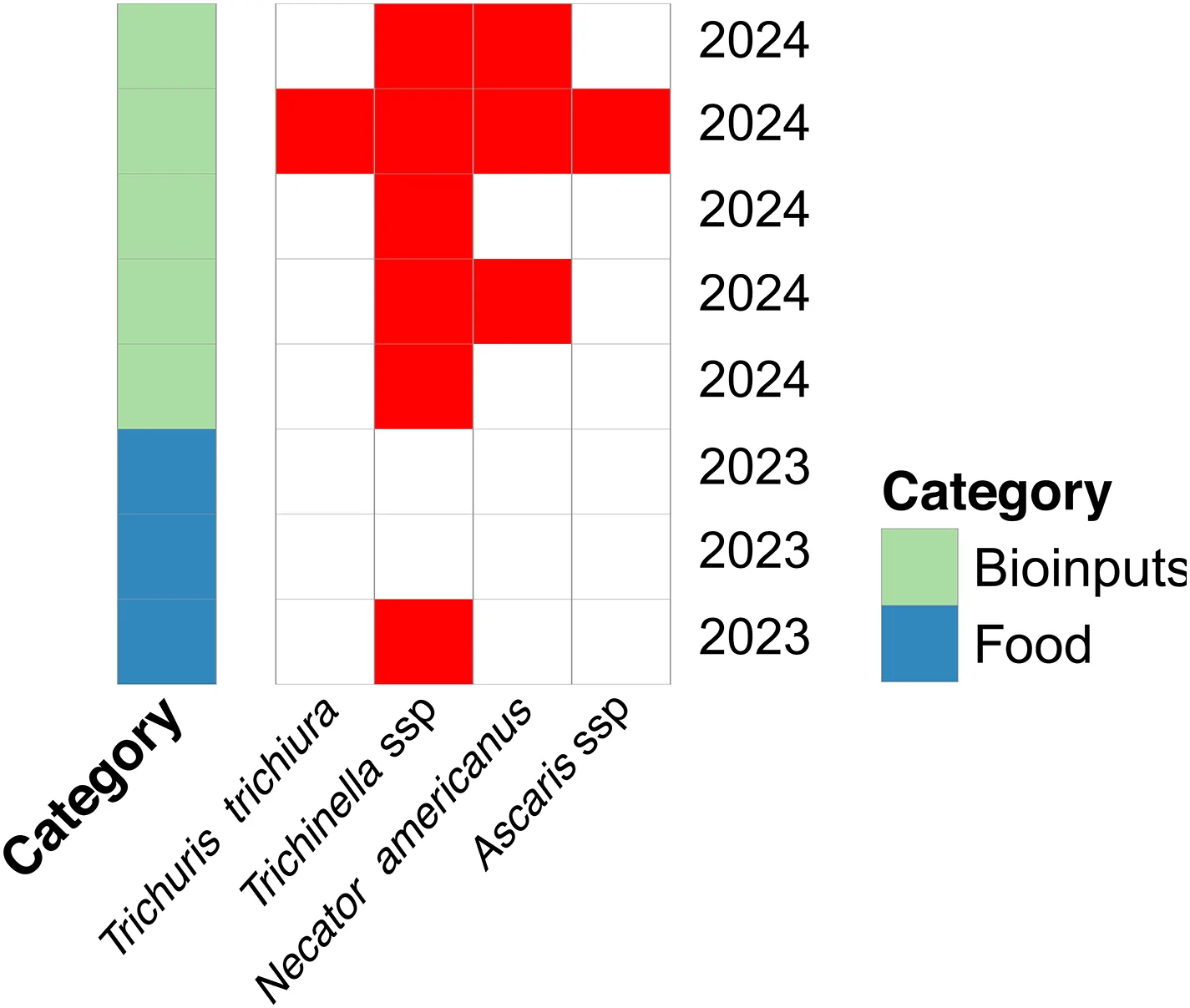
Comparative heatmaps of nematode DNA detection across bioinputs and food samples. Commercial bioinputs and food items, evidencing potential parasite transmission routes through the reuse of treated or untreated biosolids in agriculture.

Regarding the food samples analyzed in 2023, only one sample (M9A2001) contained DNA corresponding to *Trichinella* spp. (Fig 6).

## Discussion

### Wastewater-based epidemiological surveillance and relevance of nematodes as environmental markers

Wastewater-based epidemiology (WBE) has emerged as an effective approach for monitoring the circulation of infectious agents within human populations, allowing the detection of pathogens shed into urban sanitation systems and providing an indirect representation of community-level infection dynamics [18,20]. Traditionally applied to viruses, bacteria, and protozoa, WBE is increasingly recognized as a promising framework for monitoring helminths of public health importance. In this context, the application of metataxonomic approaches enables the detection of nematode DNA in complex environmental matrices such as wastewater and biosolids, providing a scalable strategy for environmental parasite surveillance.

In the present study, the detection of nematodes such as *Trichuris trichiura*, *Ascaris* spp., *Enterobius vermicularis*, and *Necator americanus* in wastewater treatment plants indicates that these systems act as environmental reservoirs of helminth DNA originating from human populations. The presence of these taxa is consistent with their known transmission routes, primarily associated with fecal contamination and inadequate sanitation [40]. The persistence of parasitic structures such as *Ascaris* and *Trichuris* eggs in wastewater systems has been widely documented due to their high environmental resistance and ability to accumulate in sludge and biosolids during treatment processes [41].

The taxa detected in this study also correspond broadly with national epidemiological records. According to the National Survey of Intestinal Parasitism in the School Population 2012–2014 (ENPI), the most frequently reported intestinal nematodes in Colombia are *Trichuris trichiura*, *Ascaris lumbricoides*, and hookworms (*Necator americanus* / *Ancylostoma duodenale*) [7]. The recurrent detection of *T. trichiura*, *Ascaris* spp., and *N. americanus* DNA in wastewater and biosolids therefore reflects the continued circulation of these geohelminths in the population and highlights their usefulness as indicators of fecal contamination and sanitation deficiencies. Notably, *A. duodenale* DNA was not detected in the analyzed samples, suggesting that in the studied regions the dominant hookworm component may correspond primarily to *N. americanus*.

Beyond these well-known soil-transmitted helminths, the detection of zoonotic taxa such as *Angiostrongylus cantonensis* and *Trichinella* spp. expands the spectrum of parasites identifiable through environmental molecular monitoring. *A. cantonensis*, an emerging zoonotic parasite in Latin America associated with human angiostrongyliasis and eosinophilic meningoencephalitis [42,43], was detected in multiple matrices in this study.

The utility of WBE for zoonotic taxa such as *A. cantonensis* and *Trichinella* spp. differs from that for the human geohelminths. Because these parasites reach the environment mainly through intermediate or reservoir hosts (gastropods and rodents) rather than through direct human faecal shedding, their detection in wastewater and sludge does not track human infection prevalence directly; rather, it signals the environmental circulation of zoonotic agents and the presence of their hosts within the sewershed. This information remains valuable for One Health surveillance, as it can flag potential exposure risks that conventional clinical monitoring overlooks. This interpretation is supported by the local epidemiological context: human angiostrongyliasis has been reported in Colombia since 1979, and *Angiostrongylus* spp. has recently been detected in the invasive snails *Lissachatina fulica* and *Cornu aspersum* in the Aburrá Valley, frequently co-occurring with rodents [43]. Nonetheless, given that the *A. cantonensis* signal in this study rested on low-abundance, single-read mOTUs, and that molecular markers do not always resolve *A. cantonensis* from the locally prevalent *A. costaricensis*, these detections should be regarded as preliminary evidence of environmental circulation requiring targeted confirmation.

Taken together, these findings indicate that wastewater treatment systems function not only as sanitation infrastructures but also as environmental observatories for pathogen circulation. In Colombia, surveillance of soil-transmitted helminths remains largely focused on clinical reporting and periodic deworming campaigns, with limited integration of environmental data into public health monitoring systems. In this context, the metataxonomic detection of nematode DNA in wastewater provides an additional layer of information that could complement existing surveillance frameworks and support more proactive approaches to parasite monitoring within a One Health perspective**.**

Importantly, the repeated sampling of the Aguas Claras WWTP in 2021 and 2023 adds a longitudinal dimension rarely available in environmental parasite surveys. The persistent detection of *Trichinella* spp. and geohelminth DNA across both years is consistent with sustained circulation rather than sporadic contamination, reinforcing the value of metataxonomic monitoring for tracking parasite dynamics over time in these communities.

### Bioinputs and food: Parasitic risks in agricultural systems

The increasing commercialization of agricultural bioinputs derived from organic residues has expanded their use as fertilizers, soil conditioners, and microbial amendments in both small-scale and industrial agricultural systems. These products, often produced from treated organic matter or recycled waste streams, are widely promoted as sustainable alternatives to conventional agrochemicals. However, when sanitary controls are insufficient or production processes are poorly standardized, bioinputs may act as vehicles for the persistence and dissemination of microorganisms and parasitic DNA across agricultural environments. In this context, the evaluation of commercially available bioinputs and their potential role in the environmental circulation of parasites becomes particularly relevant, especially when these products are applied to soils used for food production [41,44].

Geohelminths, such as *Ascaris* spp., *Trichuris trichiura*, and *Necator americanus*, possess resistant structures that allow them to persist in environmental matrices such as water, soil, and biosolids. When these residues are used as bioinputs without proper treatment, the transmission cycle can be facilitated by contaminating agricultural soils and crops intended for human or animal consumption, thereby sustaining parasitic transmission cycles [41,44,45]. This dynamic perpetuates cross-contamination between the sanitation, agricultural, and food systems, creating an ecological circuit for parasitic persistence.

Although biological and thermal treatments significantly reduce microbial loads, various studies have demonstrated the residual presence of oocysts, cysts, and parasitic DNA even after conventional purification processes, highlighting their structural resistance [44]. Therefore, the production and use of bioinputs derived from treated sludge should include advanced sanitization processes and effectiveness controls, aimed at interrupting parasite transmission cycles and reducing associated risks [41,46].

In this regard, the safe management of bioinputs requires the integration of environmental, health, and food surveillance, ensuring that the reused residues do not pose a public health risk. Likewise, food represents a secondary exposure route, arising from the use of non-sanitized bioinputs for fertilization or from contact with contaminated environmental matrices, such as irrigation water. This connection underscores the need to assess parasitic traceability throughout the agri-food chain.

Accordingly, the molecular monitoring of nematodes and other parasites in reused matrices constitutes a key tool to strengthen agricultural biosafety, protect community health, and promote sustainability in wastewater treatment and reuse systems [45].

### Reference databases and limitations for species-level identification

The technical and technological feasibility of applying metataxonomic approaches to nematodes remains an emerging challenge. Although these methodologies are well established for bacteria and fungi, their development in parasites is still in its early stages. Nevertheless, the results obtained in this study demonstrate that it is possible to overcome, at least partially, the current limitations and move toward the standardization of specific protocols for the identification of nematodes in environmental matrices.

In this study, the combined use of the hypervariable V4 region of the 18S rDNA gene and concatenated 18S–28S reference sequences provided consistent taxonomic resolution and sufficient phylogenetic support to resolve several taxa at the genus level and, in some cases, at the species level [6,11].

The selection of the 18S rDNA V4 region as the primary marker reflected the exploratory, broad-spectrum nature of environmental surveillance. Because the taxa present in each matrix are not known a priori, a marker that combines conserved priming sites with informative hypervariable regions is required to capture a wide phylogenetic range within a single amplicon [24]. The 18S rRNA gene meets this requirement and, unlike more variable spacers, is supported by extensive, taxonomically broad reference collections that enable reliable multiple-sequence alignment and phylogenetic placement across divergent nematode lineages [5,22]. In contrast, internal transcribed spacer markers such as ITS-2 provide higher intraspecific resolution and have proven powerful for quantifying species composition within defined groups, most notably the strongylid nemabiome approach [47], but their high length and sequence variability hampers alignment across the phylum, and their reference coverage remains uneven for many environmentally relevant and zoonotic nematodes. For a heterogeneous, hypothesis-free surveillance context, the 18S marker therefore offered the most favourable balance between taxonomic breadth, alignment reliability, and database support, at the cost of species-level resolution in a few closely related genera.

The construction of the local reference database, derived from complete genomes and curated ribosomal sequences, was a key component of this study and simultaneously represented one of the main methodological challenges. Although the taxonomic coverage achieved was close to 100% of the nematodes included, the limited availability of complete genomes in public databases remains a major constraint for large-scale, high-resolution phylogenetic studies [5,22], and the biases associated with species diversity coverage will remain latent until this gap is resolved. Future efforts could expand and refine local reference databases by incorporating curated ribosomal and genomic sequences available through WormBase ParaSite, a dedicated repository of helminth genomic data that is regularly cross-referenced with NCBI resources [48].

Through the strategy of annotating and extracting 18S and 28S ribosomal regions using Barrnap, more than 98% of the expected sequences were successfully recovered, generating 62 high-quality concatenated consensus sequences, which enabled the construction of a robust phylogenetic foundation. This advancement helps to address one of the most significant gaps in nematode metataxonomy: the lack of curated databases containing complete ribosomal information, which limits the taxonomic accuracy of inferences [22].

The maximum likelihood analysis allowed the identification of 25 mOTUs with strong statistical support (UFBoot and aLRT ≥ 95%), distributed across four clades and six nematode taxa. The topological congruence observed between the trees generated in this study and previously reported nematode phylogenies reinforces the robustness and reliability of the taxonomic assignments obtained [1,3,5,6]. These results support the reliability of the concatenated ribosomal marker system for resolving both deep evolutionary relationships and recent divergences [3,5].

The phylogenetic reconstruction confirmed the presence of six nematode taxa, all six of which are relevant to public health surveillance: four are human intestinal nematodes (*Trichuris trichiura, Ascaris* spp., *Necator americanus*, and *Enterobius vermicularis*) and two are zoonotic agents (*Angiostrongylus cantonensis* and *Trichinella* spp.). Among the human intestinal nematodes, *T. trichiura*, *E. vermicularis*, and *N. americanus* were identified with high support values (≥ 95%).

Despite these advances, intraspecific resolution remains limited, especially in genera such as *Ascaris* and *Trichinella*, which exhibit low genetic divergence among closely related species. In these cases, taxonomic assignment was restricted to the genus level (spp.) due to the inability to distinguish between closely related species (*A. suum/A. lumbricoides*, *T. britovi/T. pseudospiralis*). Such limitations are consistent with previous metataxonomic studies of nematodes, where the resolving power of rDNA is considered moderate, yet sufficient for genus-level identification and effective for epidemiological surveillance [12,22].

This study demonstrates that ribosomal metataxonomics, when combined with phylogenetic validation using curated reference databases, constitutes a scalable framework for detecting parasitic nematode DNA across heterogeneous environmental matrices.

### Constraints and prospects

Beyond the taxonomic-resolution limitations discussed above, several additional constraints should be considered when interpreting these results. The detection of DNA does not necessarily imply the presence of viable or infectious organisms, so results should be interpreted in an ecological context, not solely at the molecular level. A further limitation intrinsic to amplicon-based approaches is amplification bias against low-abundance templates. Primer competition and stochastic amplification in complex environmental matrices can under-represent or randomly capture rare targets, so detections supported by very few reads should be interpreted with caution. In the present dataset, several detections including all three positive samples for *Angiostrongylus cantonensis* were supported by single-read mOTUs, underscoring that low-abundance signals warrant confirmatory approaches (e.g., targeted qPCR or deeper sequencing) before epidemiological interpretation.

The aim of this study was not to estimate parasite prevalence but to evaluate the feasibility of a metataxonomic framework for environmental surveillance across heterogeneous environmental matrices. Consequently, the results should be interpreted as evidence that the framework successfully implements and validates presence–absence detection of parasitic nematodes across diverse environmental matrices, rather than as a direct measure of parasite burden in the studied populations.

Future research should integrate higher-resolution markers, as well as phylogenomic strategies that allow more precise identification of cryptic and emerging species. Likewise, coupling spatial and temporal analyses could facilitate correlations between the presence of nematode DNA and local environmental or sanitary variables, thereby strengthening the predictive capacity of wastewater-based molecular surveillance.

## Supporting information

S1 Table Sampling design and description of study sites. Wastewater treatment plants, bioinputs, and food sources, including type, region, elevation, population served, influent flow, treatment process, biosolids production, matrices collected, number of samples, and collection years.
(XLSX)

S2 Table Sample metadata and nematode DNA detection. Read counts per taxon.
(XLSX)

S1 Fig Phylogenetic analysis of Clade IV for taxonomic assignment. Maximum-likelihood phylogenetic tree of Clade IV.
(PDF)

S1 File Metataxonomic analysis pipeline and ancillary scripts. Python script implementing the automated metataxonomic pipeline (BLAST-based candidate selection, quality filtering, rDNA extraction, sequence alignment, and maximum-likelihood tree construction) compatible with MOTHUR OTU tables, together with the Python and R (dplyr/ggplot2) scripts used for descriptive statistics and heatmap generation.
(ZIP)
