# Supplementary material for: A ribosomal marker-based metataxonomic framework for environmental surveillance of nematodes of public health importance
Source: PLoS One. 2026 Aug 28;21(8):e0348689. doi: 10.1371/journal.pone.0348689 (PMC13524279; doi:10.1371/journal.pone.0348689)
Supplement: Supplementary file 1 [file pone.0348689.s003.pdf]

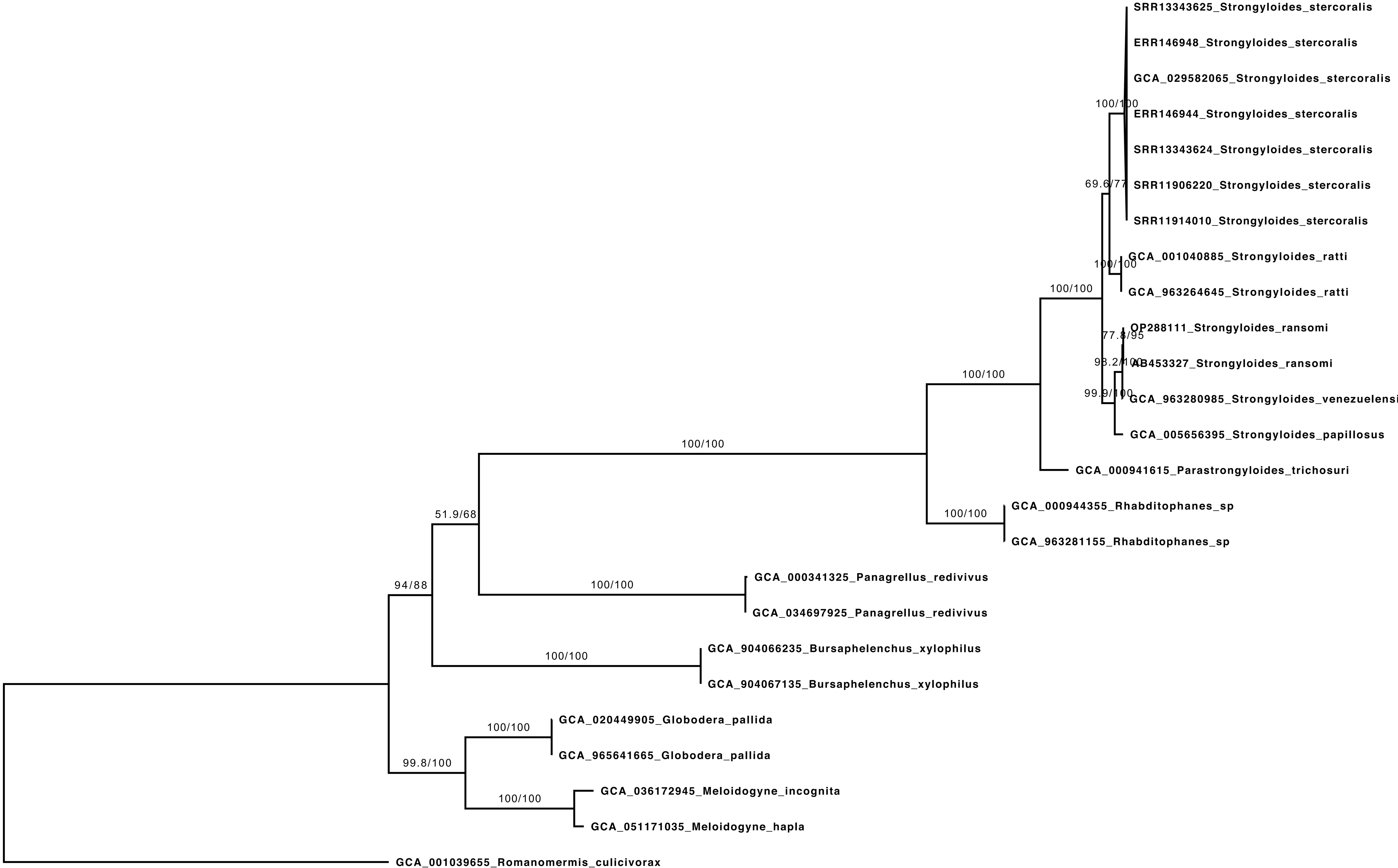
SRR13343625_Strongyloides_stercoralis
ERR146948_Strongyloides_stercoralis
GCA_029582065_Strongyloides_stercoralis
100/100
ERR146944_Strongyloides_stercoralis
SRR13343624_Strongyloides_stercoralis
69.6/77
SRR11906220_Strongyloides_stercoralis
SRR11914010_Strongyloides_stercoralis
GCA_001040885_Strongyloides_ratti
100/100
100/100
GCA_963264645_Strongyloides_ratti
OP288111_Strongyloides_ransomi
77.8/95
98.2/100
AB453327_Strongyloides_ransomi
100/100
99.9/100
GCA_963280985_Strongyloides_venezuelensis
GCA_005656395_Strongyloides_papillosus
100/100
GCA_000941615_Parastrongyloides_trichosuri
GCA_000944355_Rhabditophanes_sp
51.9/68
100/100
GCA_963281155_Rhabditophanes_sp
GCA_000341325_Panagrellus_redivivus
94/88
100/100
GCA_034697925_Panagrellus_redivivus
GCA_904066235_Bursaphelenchus_xylophilus
100/100
GCA_904067135_Bursaphelenchus_xylophilus
GCA_020449905_Globodera_pallida
100/100
99.8/100
GCA_965641665_Globodera_pallida
GCA_036172945_Meloidogyne_incognita
100/100
GCA_051171035_Meloidogyne_hapla
GCA_001039655_Romanomermis_culicivorax
