# Supplementary material for: A ribosomal marker-based metataxonomic framework for environmental surveillance of nematodes of public health importance
Source: PLoS One. 2026 Aug 28;21(8):e0348689. doi: 10.1371/journal.pone.0348689 (PMC13524279; doi:10.1371/journal.pone.0348689)
Supplement: Supplementary file 3 [file pone.0348689.s007.docx]

PONE-D-26-19237
A Ribosomal Marker-Based Metataxonomic Framework for Environmental Surveillance of Nematodes of Public Health Importance
PLOS One

Dear Dr. Alzate,

Thank you for submitting your manuscript to PLOS ONE. After careful consideration, we feel that it has merit but does not fully meet PLOS ONE’s publication criteria as it currently stands. Therefore, we invite you to submit a revised version of the manuscript that addresses the points raised during the review process.

Please submit your revised manuscript by Aug 09 2026 11:59PM. If you will need more time than this to complete your revisions, please reply to this message or contact the journal office at [plosone@plos.org](mailto:plosone@plos.org). When you're ready to submit your revision, log on to <https://www.editorialmanager.com/pone/> and select the 'Submissions Needing Revision' folder to locate your manuscript file.

Please include the following items when submitting your revised manuscript:
A letter that responds to each point raised by the academic editor and reviewer(s). You should upload this letter as a separate file labeled 'Response to Reviewers'.
A marked-up copy of your manuscript that highlights changes made to the original version. You should upload this as a separate file labeled 'Revised Manuscript with Track Changes'.
An unmarked version of your revised paper without tracked changes. You should upload this as a separate file labeled 'Manuscript'.
If you would like to make changes to your financial disclosure, please include your updated statement in your cover letter. Guidelines for resubmitting your figure files are available below the reviewer comments at the end of this letter.

If applicable, we recommend that you deposit your laboratory protocols in [protocols.io](http://protocols.io) to enhance the reproducibility of your results. Protocols.io assigns your protocol its own identifier (DOI) so that it can be cited independently in the future. For instructions see: <https://journals.plos.org/plosone/s/submission-guidelines#loc-laboratory-protocols>. Additionally, PLOS ONE offers an option for publishing peer-reviewed Lab Protocol articles, which describe protocols hosted on [protocols.io](http://protocols.io). Read more information on sharing protocols at <https://plos.org/protocols?utm_medium=editorial-email&utm_source=authorletters&utm_campaign=protocols>.

As the corresponding author, your ORCID iD is verified in the submission system and will appear in the published article. PLOS supports the use of ORCID, and we encourage all coauthors to register for an ORCID iD and use it as well. Please encourage your coauthors to verify their ORCID iD within the submission system before final acceptance, as unverified ORCID iDs will not appear in the published article. Only the individual author can complete the verification step; PLOS staff cannot verify ORCID iDs on behalf of authors.

We look forward to receiving your revised manuscript.

Kind regards,

Nils Pilotte, Ph.D.
Academic Editor
PLOS One

Journal Requirements:

When submitting your revision, we need you to address these additional requirements.

1.Please ensure that your manuscript meets PLOS ONE's style requirements, including those for file naming. The PLOS ONE style templates can be found at

<https://journals.plos.org/plosone/s/file?id=wjVg/PLOSOne_formatting_sample_main_body.pdf> and

<https://journals.plos.org/plosone/s/file?id=ba62/PLOSOne_formatting_sample_title_authors_affiliations.pdf>

2. Please note that PLOS One has specific guidelines on code sharing for submissions in which author-generated code underpins the findings in the manuscript. In these cases, we expect all author-generated code to be made available without restrictions upon publication of the work. Please review our guidelines at <https://journals.plos.org/plosone/s/materials-and-software-sharing#loc-sharing-code> and ensure that your code is shared in a way that follows best practice and facilitates reproducibility and reuse.

3. When completing the data availability statement of the submission form, you indicated that you will make your data available on acceptance. We strongly recommend all authors decide on a data sharing plan before acceptance, as the process can be lengthy and hold up publication timelines. Please note that, though access restrictions are acceptable now, your entire data will need to be made freely accessible if your manuscript is accepted for publication. This policy applies to all data except where public deposition would breach compliance with the protocol approved by your research ethics board. If you are unable to adhere to our open data policy, please kindly revise your statement to explain your reasoning and we will seek the editor's input on an exemption. Please be assured that, once you have provided your new statement, the assessment of your exemption will not hold up the peer review process.

4. We note that Figure 1 in your submission contain map/satellite images which may be copyrighted. All PLOS content is published under the Creative Commons Attribution License (CC BY 4.0), which means that the manuscript, images, and Supporting Information files will be freely available online, and any third party is permitted to access, download, copy, distribute, and use these materials in any way, even commercially, with proper attribution. For these reasons, we cannot publish previously copyrighted maps or satellite images created using proprietary data, such as Google software (Google Maps, Street View, and Earth). For more information, see our copyright guidelines: <http://journals.plos.org/plosone/s/licenses-and-copyright>.

 We require you to either (1) present written permission from the copyright holder to publish these figures specifically under the CC BY 4.0 license, or (2) remove the figures from your submission:

 1. You may seek permission from the original copyright holder of Figure 1 to publish the content specifically under the CC BY 4.0 license. 

We recommend that you contact the original copyright holder with the Content Permission Form (<http://journals.plos.org/plosone/s/file?id=7c09/content-permission-form.pdf>) and the following text:

“I request permission for the open-access journal PLOS ONE to publish XXX under the Creative Commons Attribution License (CCAL) CC BY 4.0 (<http://creativecommons.org/licenses/by/4.0/>). Please be aware that this license allows unrestricted use and distribution, even commercially, by third parties. Please reply and provide explicit written permission to publish XXX under a CC BY license and complete the attached form.”

 Please upload the completed Content Permission Form or other proof of granted permissions as an "Other" file with your submission.

 In the figure caption of the copyrighted figure, please include the following text: “Reprinted from [ref] under a CC BY license, with permission from [name of publisher], original copyright [original copyright year].”

 2. If you are unable to obtain permission from the original copyright holder to publish these figures under the CC BY 4.0 license or if the copyright holder’s requirements are incompatible with the CC BY 4.0 license, please either i) remove the figure or ii) supply a replacement figure that complies with the CC BY 4.0 license. Please check copyright information on all replacement figures and update the figure caption with source information. If applicable, please specify in the figure caption text when a figure is similar but not identical to the original image and is therefore for illustrative purposes only.

The following resources for replacing copyrighted map figures may be helpful:

 USGS National Map Viewer (public domain): <http://viewer.nationalmap.gov/viewer/>

The Gateway to Astronaut Photography of Earth (public domain): <http://eol.jsc.nasa.gov/sseop/clickmap/>

Maps at the CIA (public domain): <https://www.cia.gov/library/publications/the-world-factbook/index.html> and <https://www.cia.gov/library/publications/cia-maps-publications/index.html>

NASA Earth Observatory (public domain): <http://earthobservatory.nasa.gov/>

Landsat: <http://landsat.visibleearth.nasa.gov/>

USGS EROS (Earth Resources Observatory and Science (EROS) Center) (public domain): [http://eros.usgs.gov/#](http://eros.usgs.gov/)

Natural Earth (public domain): <http://www.naturalearthdata.com/>

5. If the reviewer comments include a recommendation to cite specific previously published works, please review and evaluate these publications to determine whether they are relevant and should be cited. There is no requirement to cite these works unless the editor has indicated otherwise. 

6. Please review your reference list to ensure that it is complete and correct. If you have cited papers that have been retracted, please include the rationale for doing so in the manuscript text, or remove these references and replace them with relevant current references. Any changes to the reference list should be mentioned in the rebuttal letter that accompanies your revised manuscript. If you need to cite a retracted article, indicate the article’s retracted status in the References list and also include a citation and full reference for the retraction notice.

Additional Editor Comments:
While the reviews of your work were extremely positive, reviewer #1 provided some worthwhile suggestions and raised a number of valid points which merit your consideration.  We look forward to receiving your revised manuscript after you have addressed these concerns.

[Note: HTML markup is below. Please do not edit.]

Reviewers' comments:

Reviewer's Responses to Questions

Comments to the Author

1. Is the manuscript technically sound, and do the data support the conclusions?

The manuscript must describe a technically sound piece of scientific research with data that supports the conclusions. Experiments must have been conducted rigorously, with appropriate controls, replication, and sample sizes. The conclusions must be drawn appropriately based on the data presented.

Reviewer #1: Yes

Reviewer #2: Yes

2. Has the statistical analysis been performed appropriately and rigorously?

Reviewer #1: Yes

Reviewer #2: Yes

3. Have the authors made all data underlying the findings in their manuscript fully available?

The PLOS Data policy requires authors to make all data underlying the findings described in their manuscript fully available without restriction, with rare exception (please refer to the Data Availability Statement in the manuscript PDF file). The data should be provided as part of the manuscript or its supporting information, or deposited to a public repository. For example, in addition to summary statistics, the data points behind means, medians and variance measures should be available. If there are restrictions on publicly sharing data—e.g. participant privacy or use of data from a third party—those must be specified.

Reviewer #1: Yes

Reviewer #2: Yes

4. Is the manuscript presented in an intelligible fashion and written in standard English?

PLOS ONE does not copyedit accepted manuscripts, so the language in submitted articles must be clear, correct, and unambiguous. Any typographical or grammatical errors should be corrected at revision, so please note any specific errors here.

Reviewer #1: Yes

Reviewer #2: Yes

5. Review Comments to the Author

Please use the space provided to explain your answers to the questions above. You may also include additional comments for the author, including concerns about dual publication, research ethics, or publication ethics. (Please upload your review as an attachment if it exceeds 20,000 characters)

Reviewer #1: Zuluaga et al. have presented novel results for nematode species-specific environmental surveillance using wastewater and agricultural biomass samples in Colombia. The methods and analyses are robust, and the manuscript has a logical flow. The study objectives were outlined and successfully executed.

The manuscript would benefit from some minor revisions as outlined below. Namely, a deeper dive into the literature for wastewater and environmental surveillance of nematodes, sequencing target selection can be addressed, and methods can be more detailed by including an in-depth description of the types of samples collected and date of collection. I have suggested a table be included to detail this information.

Introduction:
Lines 44-47: The nuance between human parasite resistance and animal infective parasite resistance should be better captured. Resistance is established in many animal infective helminths while in humans resistance may be emerging, with limited evidence supporting this claim.
Line 54: Animal and zoonotic hookworm eggs cannot be differentiated either.

• Some additional literature on other metataxonomic and wastewater/environmental surveillance for intestinal parasitic worms that should be considered:
<https://doi.org/10.1038/s41467-023-44521-3>, <https://doi.org/10.1371/journal.pntd.0012416>, <https://doi.org/10.1371/journal.pwat.0000224>, 10.1021/acs.est.1c02168, <https://doi.org/10.1371/journal>. pwat.0000337, Gilleard Nemabiome papers

Material Methods:
Were samples collected from WWTPs settled solids, influent (primary?), effluent? What time frame were samples collected? How many samples from each site were collected. A table may be useful here.

Line 85: What is the estimated population that uses each individual WWTP?
Line 90: More information on type of food samples and how these were processed as well as what the bioinput samples consist of. A breakdown of how many samples from each site and in what years they were collected would be helpful hear to interpret results (lines 233-243). Suggest including a table for this information.
122-123 Incomplete sentence here.

Results:
Full genus and species should be written out when introducing for the first time in the body of the manuscript followed by abbreviated G. species. The data here is longitudinal which should be highlighted since it makes the results more impactful and relevant for public health monitoring of parasitic spp..

235: “occasional presence”—can this be more quantitative? (x detects out of y samples or locations)

Fig1: legend mentions biofertilizers (bioinput?), should be described in methods or consistently labeled as “bioinput” or “biofertilizer”. Introducing these terms in the methods would be helpful as well. Suggest making circle around each species breakdown in the same colour as the study site.

Figure 5 / 6: presence absence data: suggest dropping sample names and rotating 90 degrees such that species names are on the x and the y is sample type and location (or category for figure 6). Remove “_” between genus and species. * / ** can be added to the right axis.

Supplemental:
Is the supplemental data the same as in the main figures? If so, there is no need for addition location-stratified supplementary figures. A table in the supplementary data of sample, location, sample type (including sludge, settled solids, influent, effluent, etc.), collection date, and detection +/- would be more fitting.

Discussion:
Address why the 18s target was chosen over other targets (ie ITS-2) either in the discussion or introduction. Sections of the discussion are repetitive and can be combined to minimize highlighting similar pitfalls (ie limited reference genomes, difficulty with species level resolution). Another pitfall that should be mentioned, however, is low abundance sequencing amplification bias using these approaches. While this is not a quantitative study, the longitudinal data coupled with species-specific detection add novel surveillance data for these Colombian communities and can be emphasized further.

298- 300: If Trichinella spp / Angiostrongylus cantonensis detection is likely from intermediate hosts, what utility does WBE have for these species? Are clinical cases rising in the sampling areas? Additional commentary on this can strengthen the argument for WBE surveillance in these communities.

357-358 More curated sequences can be obtained from WormBase ParaSite databases which is often cross referenced in NCBI and can be mentioned for future studies.

373: Mention how many out of the 6 species are relevant to public health surveillance.

395-397: “Methodological applicability” – this research implements and validates methods for presence absence detection of parasites in diverse environmental samples.

Reviewer #2: This is a well-planned study and a well-written manuscript that clearly explains the use of meta taxonomy for environmental surveillance. Rather than assessing the burden of nematodes, the manuscript presents a well-structured methodological framework that can be applied in future research.

6. PLOS authors have the option to publish the peer review history of their article (what does this mean?). If published, this will include your full peer review and any attached files.


If you choose “no”, your identity will remain anonymous but your review may still be made public.


Do you want your identity to be public for this peer review? For information about this choice, including consent withdrawal, please see our Privacy Policy.

Reviewer #1: No

Reviewer #2: No
