# Supplementary material for: A ribosomal marker-based metataxonomic framework for environmental surveillance of nematodes of public health importance
Source: PLoS One. 2026 Aug 28;21(8):e0348689. doi: 10.1371/journal.pone.0348689 (PMC13524279; doi:10.1371/journal.pone.0348689)
Supplement: Peer review history

# Peer review: Decision Letter 0

**DOI:** 10.1371/journal.pone.0348689.r001

**Authors:** Nils Pilotte

25 Jun 2026

Dear Dr. Alzate,

Thank you for submitting your manuscript to PLOS ONE. After careful consideration, we feel that it has merit but does not fully meet PLOS ONE’s publication criteria as it currently stands. Therefore, we invite you to submit a revised version of the manuscript that addresses the points raised during the review process.

Please submit your revised manuscript by Aug 09 2026 11:59PM. If you will need more time than this to complete your revisions, please reply to this message or contact the journal office at plosone@plos.org. When you're ready to submit your revision, log on to https://www.editorialmanager.com/pone/ and select the 'Submissions Needing Revision' folder to locate your manuscript file.

- A letter that responds to each point raised by the academic editor and reviewer(s). You should upload this letter as a separate file labeled 'Response to Reviewers'.
- A marked-up copy of your manuscript that highlights changes made to the original version. You should upload this as a separate file labeled 'Revised Manuscript with Track Changes'.
- An unmarked version of your revised paper without tracked changes. You should upload this as a separate file labeled 'Manuscript'.

If you would like to make changes to your financial disclosure, please include your updated statement in your cover letter. Guidelines for resubmitting your figure files are available below the reviewer comments at the end of this letter.

If applicable, we recommend that you deposit your laboratory protocols in protocols.io to enhance the reproducibility of your results. Protocols.io assigns your protocol its own identifier (DOI) so that it can be cited independently in the future. For instructions see: https://journals.plos.org/plosone/s/submission-guidelines#loc-laboratory-protocols. Additionally, PLOS ONE offers an option for publishing peer-reviewed Lab Protocol articles, which describe protocols hosted on protocols.io. Read more information on sharing protocols at https://plos.org/protocols?utm_medium=editorial-email&utm_source=authorletters&utm_campaign=protocols.

As the corresponding author, your ORCID iD is verified in the submission system and will appear in the published article. PLOS supports the use of ORCID, and we encourage all coauthors to register for an ORCID iD and use it as well. Please encourage your coauthors to verify their ORCID iD within the submission system before final acceptance, as unverified ORCID iDs will not appear in the published article. *Only*  the individual author can complete the verification step; PLOS staff *cannot*  verify ORCID iDs on behalf of authors.

We look forward to receiving your revised manuscript.

Kind regards,

Nils Pilotte, Ph.D.

Academic Editor

PLOS One

Journal Requirements:

When submitting your revision, we need you to address these additional requirements.

1.Please ensure that your manuscript meets PLOS ONE's style requirements, including those for file naming. The PLOS ONE style templates can be found at

https://journals.plos.org/plosone/s/file?id=wjVg/PLOSOne_formatting_sample_main_body.pdf and

https://journals.plos.org/plosone/s/file?id=ba62/PLOSOne_formatting_sample_title_authors_affiliations.pdf

2. Please note that PLOS One has specific guidelines on code sharing for submissions in which author-generated code underpins the findings in the manuscript. In these cases, we expect all author-generated code to be made available without restrictions upon publication of the work. Please review our guidelines at https://journals.plos.org/plosone/s/materials-and-software-sharing#loc-sharing-code and ensure that your code is shared in a way that follows best practice and facilitates reproducibility and reuse.

3. When completing the data availability statement of the submission form, you indicated that you will make your data available on acceptance. We strongly recommend all authors decide on a data sharing plan before acceptance, as the process can be lengthy and hold up publication timelines. Please note that, though access restrictions are acceptable now, your entire data will need to be made freely accessible if your manuscript is accepted for publication. This policy applies to all data except where public deposition would breach compliance with the protocol approved by your research ethics board. If you are unable to adhere to our open data policy, please kindly revise your statement to explain your reasoning and we will seek the editor's input on an exemption. Please be assured that, once you have provided your new statement, the assessment of your exemption will not hold up the peer review process.

4. We note that Figure 1 in your submission contain map/satellite images which may be copyrighted. All PLOS content is published under the Creative Commons Attribution License (CC BY 4.0), which means that the manuscript, images, and Supporting Information files will be freely available online, and any third party is permitted to access, download, copy, distribute, and use these materials in any way, even commercially, with proper attribution. For these reasons, we cannot publish previously copyrighted maps or satellite images created using proprietary data, such as Google software (Google Maps, Street View, and Earth). For more information, see our copyright guidelines: http://journals.plos.org/plosone/s/licenses-and-copyright.

We require you to either (1) present written permission from the copyright holder to publish these figures specifically under the CC BY 4.0 license, or (2) remove the figures from your submission:

1. You may seek permission from the original copyright holder of Figure 1 to publish the content specifically under the CC BY 4.0 license.

We recommend that you contact the original copyright holder with the Content Permission Form (http://journals.plos.org/plosone/s/file?id=7c09/content-permission-form.pdf) and the following text:

“I request permission for the open-access journal PLOS ONE to publish XXX under the Creative Commons Attribution License (CCAL) CC BY 4.0 (http://creativecommons.org/licenses/by/4.0/). Please be aware that this license allows unrestricted use and distribution, even commercially, by third parties. Please reply and provide explicit written permission to publish XXX under a CC BY license and complete the attached form.”

Please upload the completed Content Permission Form or other proof of granted permissions as an "Other" file with your submission.

In the figure caption of the copyrighted figure, please include the following text: “Reprinted from [ref] under a CC BY license, with permission from [name of publisher], original copyright [original copyright year].”

2. If you are unable to obtain permission from the original copyright holder to publish these figures under the CC BY 4.0 license or if the copyright holder’s requirements are incompatible with the CC BY 4.0 license, please either i) remove the figure or ii) supply a replacement figure that complies with the CC BY 4.0 license. Please check copyright information on all replacement figures and update the figure caption with source information. If applicable, please specify in the figure caption text when a figure is similar but not identical to the original image and is therefore for illustrative purposes only.

The following resources for replacing copyrighted map figures may be helpful:

USGS National Map Viewer (public domain): http://viewer.nationalmap.gov/viewer/

The Gateway to Astronaut Photography of Earth (public domain): http://eol.jsc.nasa.gov/sseop/clickmap/

Maps at the CIA (public domain): https://www.cia.gov/library/publications/the-world-factbook/index.html and https://www.cia.gov/library/publications/cia-maps-publications/index.html

NASA Earth Observatory (public domain): http://earthobservatory.nasa.gov/

Landsat: http://landsat.visibleearth.nasa.gov/

USGS EROS (Earth Resources Observatory and Science (EROS) Center) (public domain): http://eros.usgs.gov/#

Natural Earth (public domain): http://www.naturalearthdata.com/

5. If the reviewer comments include a recommendation to cite specific previously published works, please review and evaluate these publications to determine whether they are relevant and should be cited. There is no requirement to cite these works unless the editor has indicated otherwise.

6. Please review your reference list to ensure that it is complete and correct. If you have cited papers that have been retracted, please include the rationale for doing so in the manuscript text, or remove these references and replace them with relevant current references. Any changes to the reference list should be mentioned in the rebuttal letter that accompanies your revised manuscript. If you need to cite a retracted article, indicate the article’s retracted status in the References list and also include a citation and full reference for the retraction notice.

Additional Editor Comments:

While the reviews of your work were extremely positive, reviewer #1 provided some worthwhile suggestions and raised a number of valid points which merit your consideration.  We look forward to receiving your revised manuscript after you have addressed these concerns.

[Note: HTML markup is below. Please do not edit.]

Reviewers' comments:

Reviewer's Responses to Questions

**Comments to the Author**

1. Is the manuscript technically sound, and do the data support the conclusions?

Reviewer #1: Yes

Reviewer #2: Yes

**********

2. Has the statistical analysis been performed appropriately and rigorously?

Reviewer #1: Yes

Reviewer #2: Yes

**********

3. Have the authors made all data underlying the findings in their manuscript fully available?

The PLOS Data policy

Reviewer #1: Yes

Reviewer #2: Yes

**********

4. Is the manuscript presented in an intelligible fashion and written in standard English?

Reviewer #1: Yes

Reviewer #2: Yes

**********

Reviewer #1: Zuluaga et al. have presented novel results for nematode species-specific environmental surveillance using wastewater and agricultural biomass samples in Colombia. The methods and analyses are robust, and the manuscript has a logical flow. The study objectives were outlined and successfully executed.

The manuscript would benefit from some minor revisions as outlined below. Namely, a deeper dive into the literature for wastewater and environmental surveillance of nematodes, sequencing target selection can be addressed, and methods can be more detailed by including an in-depth description of the types of samples collected and date of collection. I have suggested a table be included to detail this information.

Introduction:

Lines 44-47: The nuance between human parasite resistance and animal infective parasite resistance should be better captured. Resistance is established in many animal infective helminths while in humans resistance may be emerging, with limited evidence supporting this claim.

Line 54: Animal and zoonotic hookworm eggs cannot be differentiated either.

• Some additional literature on other metataxonomic and wastewater/environmental surveillance for intestinal parasitic worms that should be considered:

https://doi.org/10.1038/s41467-023-44521-3, https://doi.org/10.1371/journal.pntd.0012416, https://doi.org/10.1371/journal.pwat.0000224, 10.1021/acs.est.1c02168, https://doi.org/10.1371/journal.pwat.0000337, Gilleard Nemabiome papers

Material Methods:

Were samples collected from WWTPs settled solids, influent (primary?), effluent? What time frame were samples collected? How many samples from each site were collected. A table may be useful here.

Line 85: What is the estimated population that uses each individual WWTP?

Line 90: More information on type of food samples and how these were processed as well as what the bioinput samples consist of. A breakdown of how many samples from each site and in what years they were collected would be helpful hear to interpret results (lines 233-243). Suggest including a table for this information.

122-123 Incomplete sentence here.

Results:

Full genus and species should be written out when introducing for the first time in the body of the manuscript followed by abbreviated G. species. The data here is longitudinal which should be highlighted since it makes the results more impactful and relevant for public health monitoring of parasitic spp..

235: “occasional presence”—can this be more quantitative? (x detects out of y samples or locations)

Fig1: legend mentions biofertilizers (bioinput?), should be described in methods or consistently labeled as “bioinput” or “biofertilizer”. Introducing these terms in the methods would be helpful as well. Suggest making circle around each species breakdown in the same colour as the study site.

Figure 5 / 6: presence absence data: suggest dropping sample names and rotating 90 degrees such that species names are on the x and the y is sample type and location (or category for figure 6). Remove “_” between genus and species. * / ** can be added to the right axis.

Supplemental:

Is the supplemental data the same as in the main figures? If so, there is no need for addition location-stratified supplementary figures. A table in the supplementary data of sample, location, sample type (including sludge, settled solids, influent, effluent, etc.), collection date, and detection +/- would be more fitting.

Discussion:

Address why the 18s target was chosen over other targets (ie ITS-2) either in the discussion or introduction. Sections of the discussion are repetitive and can be combined to minimize highlighting similar pitfalls (ie limited reference genomes, difficulty with species level resolution). Another pitfall that should be mentioned, however, is low abundance sequencing amplification bias using these approaches. While this is not a quantitative study, the longitudinal data coupled with species-specific detection add novel surveillance data for these Colombian communities and can be emphasized further.

298- 300: If Trichinella spp / Angiostrongylus cantonensis detection is likely from intermediate hosts, what utility does WBE have for these species? Are clinical cases rising in the sampling areas? Additional commentary on this can strengthen the argument for WBE surveillance in these communities.

357-358 More curated sequences can be obtained from WormBase ParaSite databases which is often cross referenced in NCBI and can be mentioned for future studies.

373: Mention how many out of the 6 species are relevant to public health surveillance.

395-397: “Methodological applicability” – this research implements and validates methods for presence absence detection of parasites in diverse environmental samples.

Reviewer #2: This is a well-planned study and a well-written manuscript that clearly explains the use of meta taxonomy for environmental surveillance. Rather than assessing the burden of nematodes, the manuscript presents a well-structured methodological framework that can be applied in future research.

**********

what does this mean?). If published, this will include your full peer review and any attached files.

If you choose “no”, your identity will remain anonymous but your review may still be made public.

**Do you want your identity to be public for this peer review?**  For information about this choice, including consent withdrawal, please see our Privacy Policy

Reviewer #1: No

Reviewer #2: No

**********

[NOTE: If reviewer comments were submitted as an attachment file, they will be attached to this email and accessible via the submission site. Please log into your account, locate the manuscript record, and check for the action link "View Attachments". If this link does not appear, there are no attachment files.]

To ensure your figures meet our technical requirements, please review our figure guidelines: https://journals.plos.org/plosone/s/figures

You may also use PLOS’s free figure tool, NAAS, to help you prepare publication quality figures: https://journals.plos.org/plosone/s/figures#loc-tools-for-figure-preparation.

NAAS will assess whether your figures meet our technical requirements by comparing each figure against our figure specifications.

Attachment Submitted filename: PONE-D-26-19237_reviewer.pdf

# Author comment: Author response to Decision Letter 1

**DOI:** 10.1371/journal.pone.0348689.r002

14 Jul 2026

Response to Reviewers

Manuscript PONE-D-26-19237

A Ribosomal Marker-Based Metataxonomic Framework for Environmental Surveillance of Nematodes of Public Health Importance

We thank the Academic Editor and both reviewers for their careful and encouraging evaluation. We were glad that all technical criteria (soundness, statistical rigour, data availability, and language) were assessed positively by both reviewers, and we are grateful to Reviewer #1 for a set of suggestions that have measurably improved the manuscript. Every point has been addressed. Below, comments are grouped by manuscript section; each is quoted, followed by our response and, where useful, the exact wording introduced in the revised text.

Note on line numbers. The line numbers below are those of the original submission, as cited by the reviewer. The precise location of each change is marked in the “Revised Manuscript with Track Changes” file. Throughout, we also made minor grammatical and stylistic corrections (punctuation, italicization of species names, and consistent use of the term “bioinput”) that are not itemized individually.

A. Response to Journal Requirements

1. Style and file naming

Response. The manuscript has been formatted to the PLOS ONE style templates, and all files have been named according to the journal's conventions (e.g., Fig1.tiff, Fig5.tiff, S1_Table, S2_Table, S1_File).

2. Code sharing

Response. All author-generated code underpinning the findings is provided, without restriction, as Supporting Information (S1 File), and will be freely available under the article's CC BY 4.0 license upon publication. S1 File contains the automated metataxonomic pipeline and the Python and R analysis/plotting scripts, making the complete analytical workflow openly reproducible.

3. Data availability

Response. We have revised our data-availability plan to comply fully with the open-data policy (updated statement in Section E). The raw amplicon sequencing reads are publicly available in the NCBI Sequence Read Archive under BioProject accession PRJNA976754; the per-sample detection data underlying every figure are provided in the new S2 Table; the sampling design is provided in the new S1 Table; and the complete analysis code is provided in S1 File.

4. Figure 1 (map/satellite imagery)

Response. Figure 1 contains no copyrighted map or satellite imagery from proprietary sources such as Google Maps, Street View, or Google Earth. The map was produced entirely by the authors in QGIS v3.40.11 from openly licensed data. The terrain relief is based on the digital elevation model distributed with the ALOS PALSAR High-Resolution Radiometrically Terrain-Corrected product of the Alaska Satellite Facility DAAC — a resampled copy of the public-domain SRTM elevation model, shared without restriction under the NASA EOSDIS Data Use Policy (ref 38) — and the administrative boundaries were obtained from the open-data repository of the Instituto Geografico Agustin Codazzi (IGAC; ref 39), released as public information under Colombian Law 1712 of 2014. These sources are equivalent to the public-domain repositories recommended in the journal's figure-replacement guidance. Source attribution has been added to the Methods and to the legend of Figure 1.

5. Suggested references

Response. We reviewed all works suggested by Reviewer #1 and incorporated the two most directly relevant to our study: Siko et al. 2025 (PLOS Water; environmental surveillance of soil-transmitted helminths in settings without networked sewerage; now ref 23, Introduction) and Avramenko et al. 2015 (the foundational strongylid “nemabiome” ITS-2 approach; now ref 47, Discussion). The remaining suggested works were evaluated but not cited, as these two best support the specific points on environmental surveillance and marker selection that the reviewer raised.

6. Reference list

Response. The reference list was reviewed for completeness and accuracy. Five references were added (Section D), of which three correspond to reviewer/editor suggestions (refs 23, 47, 48); the other two (refs 38, 39) document the open data sources of Figure 1. No retracted articles are cited.

B. Response to Reviewer #1

We thank Reviewer #1 for recognizing that “the methods and analyses are robust” and that “the study objectives were outlined and successfully executed,” and for the constructive suggestions detailed below.

Introduction

Lines 44–47 — anthelmintic resistance nuance

Reviewer comment. The nuance between human parasite resistance and animal infective parasite resistance should be better captured. Resistance is established in many animal infective helminths while in humans resistance may be emerging, with limited evidence supporting this claim.

Response. We rewrote the passage to separate the two contexts, distinguishing established resistance in animal-infective helminths from the still-limited evidence in human helminths.

Text in the revised manuscript: “Anthelmintic resistance is well established in many animal-infective helminths, where intensive drug use has selected for resistant populations and compromised veterinary control programs (8,9). In human helminths, by contrast, resistance is not yet firmly established: reduced or suboptimal treatment responses have been reported, but the supporting evidence remains limited (10).”

Line 54 — hookworm eggs

Reviewer comment. Animal and zoonotic hookworm eggs cannot be differentiated either.

Response. We extended the example so that it now also covers animal and zoonotic hookworms, which are morphologically indistinguishable from the human species at the egg stage — species that are, moreover, represented in our reference tree (Fig 4).

Text in the revised manuscript: “…microscopic examination of hookworm eggs cannot differentiate Necator americanus from Ancylostoma duodenale, nor can it distinguish these human hookworms from the morphologically indistinguishable eggs of animal and zoonotic hookworm species such as Ancylostoma ceylanicum and Ancylostoma caninum (14).”

Additional literature on environmental surveillance

Reviewer comment. Some additional literature on other metataxonomic and wastewater/environmental surveillance for intestinal parasitic worms that should be considered: [https://doi.org/10.1038/s41467-023-44521-3; https://doi.org/10.1371/journal.pntd.0012416; https://doi.org/10.1371/journal.pwat.0000224; 10.1021/acs.est.1c02168; https://doi.org/10.1371/journal.pwat.0000337], Gilleard Nemabiome papers.

Response. We incorporated the two most pertinent works. Siko et al. 2025 (ref 23) was added to the Introduction to situate our study within the growing body of environmental surveillance of soil-transmitted helminths; the Gilleard-group nemabiome reference (Avramenko et al. 2015, ref 47) was added to the Discussion to support the 18S-versus-ITS-2 comparison.

Text in the revised manuscript: “In parallel, wastewater- and soil-based environmental surveillance is increasingly being explored as a complementary strategy for monitoring soil-transmitted helminths in endemic settings (23).”

Materials and Methods

Sample matrices, timeframe, and number of samples per site (and Line 85, population per WWTP)

Reviewer comment. Were samples collected from WWTPs settled solids, influent (primary?), effluent? What time frame were samples collected? How many samples from each site were collected. A table may be useful here. [Line 85] What is the estimated population that uses each individual WWTP?

Response. We substantially expanded the Sample Collection subsection. Each sample is now attributable to a defined matrix (influent water, biosolid, effluent water, or residual sludge); the treatment train of each plant is specified (Cañaveralejo applies advanced primary treatment, the other three use activated sludge); the population served, influent flow, elevation, and daily biosolids output are given per plant; and the sampling years are stated. As suggested, we added S1 Table summarizing the full sampling design for every site.

Text in the revised manuscript: “San Fernando treats an influent of ~1.3 m³/s of municipal wastewater by activated sludge, serving 700,000 inhabitants … Aguas Claras treats ~5 m³/s by activated sludge, serving 2,200,000 inhabitants … Cañaveralejo, in the city of Cali, applies advanced primary treatment to ~4 m³/s … serves 2,600,000 inhabitants … El Retiro is a smaller rural plant … serves 20,000 inhabitants … Sampling design, sample types, and collection years for all sites are summarized in S1 Table.”

Line 90 — food and bioinput description

Reviewer comment. More information on type of food samples and how these were processed as well as what the bioinput samples consist of.

Response. We added a generic description of both matrix types. As these were commercial products, their specific brands and formulations are proprietary and cannot be disclosed; this is now stated explicitly. All samples were processed with the same extraction protocol.

Text in the revised manuscript: “Five commercial bioinput samples, solid and liquid organic fertilizers marketed for agricultural use (three from Medellín and two from Cali) and three food samples from the Aburrá Valley, comprising fresh produce intended for raw consumption and a processed cereal-based product, were analysed. As these were commercially available products, their specific formulations and brands are proprietary and are not disclosed.”

Lines 122–123 — incomplete sentence

Reviewer comment. Incomplete sentence here.

Response. The sentence was completed.

Text in the revised manuscript: “For this analysis, nematodes of public health importance were selected, including human-parasitic and zoonotic species (27,28).”

Results

Full genus/species at first mention; longitudinal emphasis

Reviewer comment. Full genus and species should be written out when introducing for the first time in the body of the manuscript followed by abbreviated G. species. The data here is longitudinal which should be highlighted since it makes the results more impactful and relevant for public health monitoring.

Response. All taxa are now written in full at first mention in the Results and abbreviated thereafter. We also made the longitudinal design explicit: the Aguas Claras plant, sampled in 2021 and 2023, is now framed as a two-year series, and a dedicated Discussion paragraph underscores the value of this longitudinal signal for public-health monitoring.

Text in the revised manuscript: “Aguas Claras WWTP, sampled in both 2021 and 2023, showed a consistent longitudinal pattern of detection … Overall, Trichinella spp. was recovered from every Aguas Claras sample, and geohelminth DNA (N. americanus and Ascaris spp.) was detected in both sampling years, indicating sustained circulation rather than sporadic contamination.”

Line 235 — “occasional presence”

Reviewer comment. “occasional presence”—can this be more quantitative? (x detects out of y samples or locations)

Response. We replaced the qualitative phrasing with an explicit detection frequency, and the per-species frequencies across all 20 samples are now available in S2 Table (Trichinella spp., 14/20; N. americanus, 7/20; Ascaris spp., 4/20; A. cantonensis, 3/20; T. trichiura, 2/20; E. vermicularis, 1/20).

Text in the revised manuscript: “Nematode DNA was detected in 10 of the 12 WWTP samples, with defined spatial distribution patterns at each study site (Fig 5).”

Figures

Figure 1 — terminology and site-coloured circles

Reviewer comment. Fig1: legend mentions biofertilizers (bioinput?), should be described in methods or consistently labeled as “bioinput” or “biofertilizer”. Introducing these terms in the methods would be helpful as well. Suggest making circle around each species breakdown in the same colour as the study site.

Response. We standardized the terminology to “bioinput” throughout the manuscript, figures, and legends, and introduced the term in the Methods. Figure 1 was redrawn so that each species-breakdown pie is now outlined in the colour assigned to its study site, visually linking every pie to its location.

Figures 5 and 6 — layout

Reviewer comment. Presence absence data: suggest dropping sample names and rotating 90 degrees such that species names are on the x and the y is sample type and location (or category for figure 6). Remove “_” between genus and species. * / ** can be added to the right axis.

Response. Both figures were redrawn exactly as suggested: rotated 90° so that species names are on the x-axis and sample type/location (Fig 5) or category (Fig 6) on the y-axis; the sample codes were removed; the underscores between genus and species were removed and species names set in italics. In place of the asterisk notation, we display the sample collection dates explicitly along the right axis, which we felt made the temporal information clearer.

Supporting information

Redundant location-stratified figures

Reviewer comment. Is the supplemental data the same as in the main figures? If so, there is no need for additional location-stratified supplementary figures. A table … of sample, location, sample type … collection date, and detection +/- would be more fitting.

Response. We agree, and we followed this recommendation. The location-stratified supplementary figures, which duplicated Figure 5, were removed. In their place we provide S2 Table (sample, category, site, region, sample type, collection year, and detection expressed as read counts per taxon) and S1 Table (sampling design). S1 Fig (Clade IV phylogeny) is retained because it conveys information not shown elsewhere.

Discussion

18S versus ITS-2 target selection

Reviewer comment. Address why the 18s target was chosen over other targets (ie ITS-2) either in the discussion or introduction.

Response. We added a paragraph justifying the marker choice. Because the taxa present in an environmental matrix are not known a priori, a broad-range marker with conserved priming sites, informative hypervariable regions, and extensive well-aligned reference coverage is required; the 18S rDNA V4 region meets these criteria. We contrast it with ITS-2, which yields higher intraspecific resolution within defined groups (the nemabiome approach) but is difficult to align across the phylum and has uneven reference coverage for zoonotic taxa.

Text in the revised manuscript: “…internal transcribed spacer markers such as ITS-2 provide higher intraspecific resolution and have proven powerful for quantifying species composition within defined groups, most notably the strongylid “nemabiome” approach (47), but their high length and sequence variability hampers alignment across the phylum, and their reference coverage remains uneven for many environmentally relevant and zoonotic nematodes.”

Repetitive sections

Reviewer comment. Sections of the discussion are repetitive and can be combined to minimize highlighting similar pitfalls (ie limited reference genomes, difficulty with species level resolution).

Response. We consolidated these points. The intraspecific-resolution and limited-reference-genome limitations are now discussed once, and the “Constraints and prospects” section opens by referring back to them rather than restating them.

Text in the revised manuscript: “Beyond the taxonomic-resolution limitations discussed above, several additional constraints should be considered when interpreting these results.”

Low-abundance amplification bias

Reviewer comment. Another pitfall that should be mentioned, however, is low abundance sequencing amplification bias using these approaches.

Response. We added this limitation and, in the spirit of transparency, tied it to our own data: several detections — including all three positive samples for Angiostrongylus cantonensis — rested on single-read mOTUs and therefore require orthogonal confirmation.

Text in the revised manuscript: “A further limitation intrinsic to amplicon-based approaches is amplification bias against low-abundance templates … several detections including all three positive samples for Angiostrongylus cantonensis were supported by single-read mOTUs, underscoring that low-abundance signals warrant confirmatory approaches (e.g., targeted qPCR or deeper sequencing) before epidemiological interpretation.”

Lines 298–300 — WBE utility for zoonotic taxa

Reviewer comment

Attachment Submitted filename: Rebuttal_letter.docx

# Peer review: Decision Letter 1

**DOI:** 10.1371/journal.pone.0348689.r003

**Authors:** Nils Pilotte

11 Aug 2026

A Ribosomal Marker-Based Metataxonomic Framework for Environmental Surveillance of Nematodes of Public Health Importance

PONE-D-26-19237R1

Dear Dr. Alzate,

We’re pleased to inform you that your manuscript has been judged scientifically suitable for publication and will be formally accepted for publication once it meets all outstanding technical requirements.

Within one week, you’ll receive an e-mail detailing the required amendments. When these have been addressed, you’ll receive a formal acceptance letter and your manuscript will be scheduled for publication.

An invoice will be generated when your article is formally accepted. Please note, if your institution has a publishing partnership with PLOS and your article meets the relevant criteria, all or part of your publication costs will be covered. Please make sure your user information is up-to-date by logging into Editorial Manager at Editorial Manager® and clicking the ‘Update My Information' link at the top of the page. For questions related to billing, please contact billing support.

If your institution or institutions have a press office, please notify them about your upcoming paper to help maximize its impact. If they’ll be preparing press materials, please inform our press team as soon as possible -- no later than 48 hours after receiving the formal acceptance. Your manuscript will remain under strict press embargo until 2 pm Eastern Time on the date of publication. For more information, please contact onepress@plos.org.

Kind regards,

Nils Pilotte, Ph.D.

Academic Editor

PLOS One

Additional Editor Comments (optional):

Reviewers' comments:

Reviewer's Responses to Questions

**Comments to the Author**

Reviewer #1: All comments have been addressed

**********

2. Is the manuscript technically sound, and do the data support the conclusions?

Reviewer #1: Yes

**********

3. Has the statistical analysis been performed appropriately and rigorously?

Reviewer #1: Yes

**********

4. Have the authors made all data underlying the findings in their manuscript fully available?

The PLOS Data policy

Reviewer #1: Yes

**********

5. Is the manuscript presented in an intelligible fashion and written in standard English?

Reviewer #1: Yes

**********

Reviewer #1: (No Response)

**********

what does this mean?). If published, this will include your full peer review and any attached files.

If you choose “no”, your identity will remain anonymous but your review may still be made public.

**Do you want your identity to be public for this peer review?**  For information about this choice, including consent withdrawal, please see our Privacy Policy

Reviewer #1: No

**********

# Editor report: Acceptance letter

**DOI:** 10.1371/journal.pone.0348689.r004

**Authors:** Nils Pilotte

PONE-D-26-19237R1

PLOS One

Dear Dr. Alzate,

I'm pleased to inform you that your manuscript has been deemed suitable for publication in PLOS One. Congratulations! Your manuscript is now being handed over to our production team.

At this stage, our production department will prepare your paper for publication. This includes ensuring the following:

* All references, tables, and figures are properly cited

* All relevant supporting information is included in the manuscript submission,

* There are no issues that prevent the paper from being properly typeset

You will receive further instructions from the production team, including instructions on how to review your proof when it is ready. Please keep in mind that we are working through a large volume of accepted articles, so please give us a few days to review your paper and let you know the next and final steps.

Lastly, if your institution or institutions have a press office, please let them know about your upcoming paper now to help maximize its impact. If they'll be preparing press materials, please inform our press team within the next 48 hours. Your manuscript will remain under strict press embargo until 2 pm Eastern Time on the date of publication. For more information, please contact onepress@plos.org.

You will receive an invoice from PLOS for your publication fee after your manuscript has reached the completed accept phase. If you receive an email requesting payment before acceptance or for any other service, this may be a phishing scheme. Learn how to identify phishing emails and protect your accounts at https://explore.plos.org/phishing.

If we can help with anything else, please email us at customercare@plos.org.

Thank you for submitting your work to PLOS One and supporting open access.

Kind regards,

PLOS ONE Editorial Office Staff

on behalf of

Dr. Nils Pilotte

Academic Editor

PLOS One
